# *Bifidobacterium infantis* Metabolizes 2′Fucosyllactose-Derived and Free Fucose Through a Common Catabolic Pathway Resulting in 1,2-Propanediol Secretion

**DOI:** 10.3389/fnut.2020.583397

**Published:** 2020-11-24

**Authors:** Liv R. Dedon, Ezgi Özcan, Asha Rani, David A. Sela

**Affiliations:** ^1^Department of Food Science, University of Massachusetts Amherst, Amherst, MA, United States; ^2^Department of Microbiology, University of Massachusetts Amherst, Amherst, MA, United States; ^3^Department of Microbiology and Physiological Systems and Center for Microbiome Research, University of Massachusetts Medical School, Worcester, MA, United States

**Keywords:** bifidobacteria, human milk oligosaccharides, fucose, 2′fucosyllactose, microbiota, microbiome

## Abstract

Human milk oligosaccharides (HMOs) enrich beneficial bifidobacteria in the infant gut microbiome which produce molecules that impact development and physiology. 2′fucosyllactose (2′FL) is a highly abundant fucosylated HMO which is utilized by *Bifidobacterium longum* subsp. *infantis*, despite limited scientific understanding of the underlying mechanism. Moreover, there is not a current consensus on whether free fucose could be metabolized when not incorporated in a larger oligosaccharide structure. Based on metabolic and genomic analyses, we hypothesize that *B. infantis* catabolizes both free fucose and fucosyl oligosaccharide residues to produce 1,2-propanediol (1,2-PD). Accordingly, systems-level approaches including transcriptomics and proteomics support this metabolic path. Co-fermentation of fucose and limiting lactose or glucose was found to promote significantly higher biomass and 1,2-PD concentrations than individual substrates, suggesting a synergistic effect. In addition, and during growth on 2′FL, *B. infantis* achieves significantly higher biomass corresponding to increased 1,2-PD. These findings support a singular fucose catabolic pathway in *B. infantis* that is active on both free and HMO-derived fucose and intimately linked with central metabolism. The impact of fucose and 2′FL metabolism on *B. infantis* physiology provides insight into the role of fucosylated HMOs in influencing host- and microbe-microbe interactions within the infant gut microbiome.

## Introduction

Human milk nourishes infants and contains host-indigestible human milk oligosaccharides (HMOs) in addition to bioavailable nutrients. These compounds contribute to overall infant nutrition by shifting gut microbiome composition to favor beneficial microbiota that metabolize HMOs to bioactive products (1–5). Despite incorporating the same monosaccharide building blocks, HMOs vary structurally at the level of primary sequence and are diversified by degree of polymerization, branching, and secondary modifications such as fucosylation (6). 2′ Fucosyllactose (2′FL) is the most abundant fucosylated HMO and accounts for up to 30% of total HMOs in secretor women (6, 7). 2′FL has been designated generally recognized as safe by the U.S. Food and Drug Administration as well as obtaining similar status by other regulatory agencies. As a consequence, it is added to commercial infant formula to more closely mimic the composition of human milk.

*Bifidobacterium longum* subsp. *infantis* (*B. infantis*) is often the dominant microbe in the infant gut microbiome where it utilizes fucosylated HMOs such as 2′FL. Several studies report that *B. infantis* utilizes 2′FL as a sole carbohydrate source (8–11). The metabolic fate of the fucosyl moiety of 2′FL, however, remains uncertain in its intracellular catabolism. Mechanistic hypotheses based solely on genomics are hindered due to the absence of identifiable fucose-active genes. Specifically, *B. infantis* lacks the full complement of the canonical pathway genes possessed by other bacteria such as *Escherichia coli* (12–14). This catabolic pathway results in the production of 1,2-propanediol (1,2-PD) through a series of phosphorylated intermediates (15, 16). Briefly, the pathway is regulated by *fucR* and relies on a fucose permease (*fucP*) to import fucose intracellularly where it is subsequently catabolized. Fucose isomerase (*fucI*) converts L-fucose to L-fuculose, which is subsequently phosphorylated by fucose kinase (*fucK*). L-fuconate-1-phosphate is converted to dihydroxyacetone phosphate and lactaldehyde by an aldolase (*fucA*). Dihydroxyacetone phosphate can subsequently be shunted to the central glycolytic pathway and lactaldehyde converted to 1,2-PD by an oxidoreductase (*fucO*) under anaerobic conditions (17). *B. infantis* ATCC 15697^T^ lacks the majority of homologous genes predicted in this pathway, although the genome encodes *fucO* which could produce 1,2-PD from lactaldehyde. Despite this, *B. infantis* has been previously observed to metabolize free fucose and produces 1,2-PD in co-fermentation with a low concentration of glucose (14).

Other bacteria such as *Xanthomonas campestris* and *Campylobacter jejuni* catabolize fucose through phosphorylation-independent pathways to produce lactate as an end product rather than 1,2-PD (12, 13) Moreover, *Sphingomonas* sp. metabolizes rhamnose, an isomer of fucose, to produce lactadehyde and pyruvate (18). The pathway requires a permease prior to intracellular conversion of fucose to fuconolactone by fucose dehydrogenase, and then hydrolyzed to form fuconate by fuconolactone hydrolase. Fuconate dehydratase then catalyzes the production of 2-keto-3-deoxy-fuconate, which is further dehydrogenated to 2,4-diketo-3-deoxy-fuconate. The metabolic fate of 2,4-diketo-3-deoxy-fuconate diverges in that *X. campestris* produces lactate and pyruvate by a hydrolysis reaction whereas *Sphingomonas* sp. produces lactaldehyde and pyruvate by an aldol reaction. Homologous genes to those used in these pathways are identifiable in the *B. infantis* genome. The presence of these putative fucose genes informed a hypothetical bifidobacterial fucose metabolic pathway in *B. infantis* and other infant-associated bifidobacteria (11, 14).

Studies of bifidobacterial fucose metabolism have generally focused on fucosylated HMOs such as 2′FL. Identification of homologous genes, functional genomic analyses such as transcriptomics, and quantitation of metabolic end products 1,2-PD and short-chain fatty acids have heretofore provided the primary evidence for the bifidobacterial fucose metabolic pathway (11, 14, 19). Thus the mechanism underlying bifidobacterial fucose metabolism remains hypothetical in the absence of direct observation of metabolic intermediates and loss-of-function genetic perturbations. Furthermore, *B. infantis* growth on free fucose as a sole carbohydrate source is incompletely characterized as existing studies focus on demonstrating *B. infantis* growth on free fucose or in co-fermentation (14, 20–22). As such, much uncertainty remains in the path by which *B. infantis* utilizes fucose as a free product or integrated into HMOs.

This study investigates *B. infantis* utilization of 2′FL-derived and free fucose as well as the impact of carbohydrate source and concentration on fucose metabolism. In order to explicate *B. infantis* physiology while metabolizing fucose, our experimental system quantifies growth kinetics, secreted end products, the proteome, and transcriptome. As a consequence, an integrated systems-level resolution of *B. infantis* utilization of 2′FL and fucose is presented herein.

## Materials and Methods

### Bacterial Strains and Propagation

*B. longum* subsp. *infantis* strain ATCC 15697^T^ (originally isolated from human infant feces) was used in this study. Propagation was performed in de Mann, Rogosa and Sharpe (MRS; BD Difco, USA) supplemented with 0.05% (wt/vol) L-cysteine hydrochloride (Sigma-Aldrich, USA) at 37°C under anaerobic conditions (7% H_2_, 10% CO_2_, N_2_ balance) (Coy Lab, USA).

### Microplate Growth Assay

Growth phenotypes were analyzed in a 96-well format using a PowerWave HT Microplate Spectrophotometer (BioTek, USA). Overnight cultures were inoculated 1% (vol/vol) to modified MRS media (mMRS) that contains a defined carbohydrate substrate without acetate and Tween80. Carbohydrates include glucose, lactose, galactose, (Sigma-Aldrich, USA), fucose (Chem-Impex, USA), 2′fucosyllactose (BASF, Germany), and mixtures thereof at varying final concentrations (wt/vol) as the sole carbohydrate source(s). The growth assay was conducted anaerobically at 37°C until bacterial growth as measured by optical density at 600 nm (OD_600_) reached stationary phase. Each carbohydrate source or mixture of sources were evaluated in biological triplicates with three technical replicates. Inoculated mMRS without carbohydrate served as the negative control, and inoculated mMRS with 2% (wt/vol) lactose served as the positive control. Bacterial growth kinetics were calculated using Wolfram Mathematica with the equation described by Dai et al., $\Delta OD\left(t\right)=\;\Delta {OD}_{asym}\left\{\frac{1}{1+\exp\left[k\left(t_{c}-t\right)\right]}-\frac{1}{1+\exp\left[kt_{c}\right]}\right\}\;$where Δ*OD* corresponds to the adjusted optical density, Δ*OD*_*asym*_ the optical density at stationary phase, *k* the growth rate, and *t*_*c*_ the time to reach maximum growth rate (23). The growth assay was performed for 72 h at 37°C under anaerobic conditions by shaking for 5 s and assessing OD_600_ at 15-min intervals with triplicate biological and technical replicates of each broth composition. At critical points during bacterial fermentation, three technical replicates were selected at random and reserved for intracellular metabolite analysis.

Bacterial metabolism was quenched with a solution of 900 μL 60% methanol (Thermo Fisher Scientific, USA) with 0.65% (w/v) ammonium carbonate (Honeywell, USA) at −20°C added to 300 μL cell-free growth media (24, 25) The solution was thoroughly mixed and incubated 30 min at −20°C prior to pelleting cells and reserving supernatant. Cell pellet was washed with 900 μL methanol/ammonium carbonate solution at −20°C. Supernatant was reserved and diluted with 900 μL HPLC-grade water (Thermo Fisher Scientific, Waltham, MA) at 4°C. Supernatant solution was thoroughly mixed prior to storage at −80°C. Reserved pellets were resuspended in 500 μL methanol with 24 mg/L adonitol (Sigma-Aldrich, St. Louis, MO) and stored at −80°C.

### Growth Inhibition Assay

Overnight cultures were inoculated 1% (vol/vol) to MRS supplemented with 0.05% (wt/vol) L-cysteine hydrochloride (Sigma-Aldrich, USA) modified with added 1,2-PD, lactate, or phosphate-buffered saline. Each condition was evaluated in biological triplicate. Lactate was used as a control as it is known to inhibit *B. infantis* growth. MRS and equal volumes of phosphate-buffered saline served as additional controls. 1,2-PD concentration ranged from 0.08 to 168 mM and lactate concentration ranged between 0.07 to 144 mM. Cultures were grown anaerobically at 37°C for 24 h after which bacterial growth was measured by OD_600_. Inhibition was determined using a comparison of the final OD_600_ at each concentration as compared to other concentrations and controls.

### Characterization of Microbial Metabolic Intermediate and End Products

Bacterial growth for metabolic profiling was performed in 15 replicates of 2 mL mMRS inoculated at a concentration of 1% (v/v) and incubated for 48 h at 37°C under anaerobic conditions. OD_600_ was assessed using a NanoDrop spectrophotometer (Thermo Fisher Scientific, USA). Harvested cells were pelleted, washed with 1.5 mL 1 × phosphate-buffered saline (PBS), and resuspended in 1 mL RNAlater (Sigma-Aldrich, St. Louis, MO). Cells resuspended in RNAlater were incubated for 8 h at 4°C prior to storage at −80°C. Cell-free spent growth media (500 μL) was reserved and quenched as described above and stored at −80°C.

Intracellular metabolites were extracted and derivatized for GC-MS analysis. Suspended pellets were thawed to −20°C, transferred to 2 mL tubes containing 600 mg 100 μm diameter silica lysis matrix (OPS Diagnostics, USA), and subjected to bead beating (3 × {40 s at 6.0 m/s followed by 5 min incubation at −20°C}) using a FastPrep 24 bead beater (MP Biomedicals, USA). Whole-cell lysate was transferred to a 2 mL Safe-Lock tube (Eppendorf, USA) and lysing matrix was washed with 500 μL methanol. 1 mL chloroform (Thermo Fisher Scientific, USA) was added to the pooled whole-cell lysate and methanol wash and vortexed 2 min. Samples were incubated 4 h at −20°C and vortexed for 1 min at 1-hour intervals. Debris was pelleted at 21,200 × g for 5 min in a 4°C rotor, supernatant was transferred to a clean 2 mL Safe-Lock tube and dried overnight using a Vacufuge (Eppendorf, USA) in V-AL mode at room temperature with rotation. Dried extracts were derivatized following a modified protocol (25, 26). 20 μL pyridine (Sigma-Aldrich, USA) with 20 mg/mL methoxyamine hydrochloride (Sigma-Aldrich, USA) was added to dried extracts and vortexed 2 min prior to 90 min incubation at 32°C with continuous mixing. Thirty-two μL *N-*methayl-*N*-(trimethylsilyl)trifluoroacetamide (Sigma-Aldrich, USA) was added and samples were vortexed 2 min prior to incubation (30 min at 37°C, 60 min at 32°C, and 60 min at room temperature) with constant mixing. Debris was pelleted at 17,000 × g for 5 min. 50 μL supernatant was transferred to a vial insert and stored at −80°C overnight prior to measurement. 24 mg/L standards (pyruvate, lactate, 1,2-propanediol, *L*-fucono-1,4-lactone) (Sigma-Aldrich, USA) in methanol were derivatized following the above protocol. GC-MS analysis was performed at the University of Massachusetts Mass Spectrometry Center using an Agilent 6890/5973 GC- MS (Agilent Technologies, USA). In summary, 1 μL of the derivatized sample was injected in split mode (10:1). Gas chromatography was performed on a 30 m, 0.25 mm, 0.25 μm film thickness DB-5ms column (J&W Scientific, USA) using 1.2 mL min^−1^ helium flow. Ionization of analytes was done by electron ionization (EI +) at 70 eV. After 2 min at 70°C, the temperature was increased to 200°C at 12°C s^−1^, then to 250°C at 8°C s^−1^, and to 325°C at 12°C s^−1^, followed by an additional constant temperature period of 5 min at 325°C. The transfer line temperature was set to 290°C. Full scan mass spectra were obtained from 45 to 600 m/z with 2 scans s^−1^ and a solvent delay time of 4 min. Compounds were identified using Agilent MSD ChemStation Software (Version E02.02, Agilent Technologies, USA) and NIST Spectral Search Program with NIST Standard Reference Database (Version 2.2, NIST, USA).

Extracellular metabolites were quantitated by HPLC. One mL quenched cell-free spent growth media concentrated using a Vacufuge for 4 h in V-AL mode at room temperature with rotation. Dehydrated solutes were resuspended in 200 μL HPLC-grade water and filtered through a 0.22-μm filter (Sartorius, USA) and stored at −80°C until analysis. Metabolite (2′fucosyllactose, fucose, lactose, glucose, 1,2-propanediol, acetate, lactate, and formate) concentrations were quantified using an 1260 Infinity HPLC system (Agilent Technologies, USA) equipped with a Optilab T-rEX refractive-index detector (Wyatt Technology, USA). Separation was performed using an Aminex HPX-87H column (ID 7.8 mm by 300 mm; Bio-Rad Laboratories, USA) at 30°C in a mobile phase of 5 mM H_2_SO_4_ with a flow rate of 0.6 mL/min and an injection volume of 20 μL. Analytical standards of 2′FL (BASF, Germany), fucose, lactose, glucose, organic acids (Sigma-Aldrich, USA), and 1,2-propanediol (Alfa Aesar, USA) were used to generate a standard curve from seven concentrations. Each measurement was performed in duplicate.

### Proteome Sample Preparation

Cultures were harvested at mid-exponential phase and the pellet was washed 3X with PBS buffer and stored at −80°C until subsequent analysis. Cell pellets were suspended in 1X SDS lysis and solubilization buffer (Protifi LLC, USA) and were sonicated with Bioruptor pico (Diagenode, USA) at high power for 10–15 min (sonication cycle: 30 s ON, 30 s OFF). The cell lysate was clarified by centrifugation at 13,000 g for 8 min. Protein concentrations were measured using the Pierce BCA protein Assay kit (Thermo Fisher Scientific, USA) on a Nanodrop spectrophotometer. Sample preparation for mass spectrometry was performed using the S-Trap micro universal MS sample prep kit (Protifi LLC, USA). Samples were reduced, alkylated, and trypsin digested according to manufacturer instructions. Briefly, 30–40 μg protein was loaded on S-Trap micro spin columns and washed with wash buffer. Each sample was mixed with digestion buffer containing mass spectrometry grade trypsin (Promega, USA) at a 1:50 ratio (wt/wt) and was added directly to the micro spin column. Samples were incubated in a water bath at 47°C for 1 h followed by overnight incubation at 37°C. Peptides were eluted by serial additions of 50 mM TEAB, 0.2% formate, and 0.2% formate in 50% acetonitrile. Eluted peptides were pooled and evaporated in a Vacufuge to dehydration and stored at −80°C until subsequent analysis. Tryptic peptides were purified with ZIPTIP® C18 pipette tips (Merck Millipore, USA) as per manufacturer instructions prior to MS/MS analysis.

### Mass Spectrometry Proteomics

Tryptic digests were analyzed using a Thermo Easy-nLC 1000 nanoLC system coupled to a Thermo Orbitrap Fusion mass spectrometer (Thermo Fisher Scientific, USA). Peptides were separated using a 90-min gradient of increasing concentration of acetonitrile and infused into the mass spectrometer at 300 nl/min. MS1 data was acquired at 60,000 resolution in the Orbitrap and MS/MS data were acquired in a data-dependent manner selecting the top five most intense ions for fragmentation, using a nominal CID collision energy of 35%. Data were processed using Proteome Discoverer 2.0 (Thermo Fisher Scientific, USA). MS/MS data was queried against a database constructed from the UniProt database (www.uniprot.org) containing *Bifidobacterium longum* subsp. *infantis* ATCC 15697 (https://www.uniprot.org/proteomes/UP000001360, Proteome ID: UP000001360). Proteomes were analyzed using SEQUEST (27). Proteome Discoverer 2.3.0.523 (Thermo Fisher Scientific, USA). SEQUEST was searched with a fragment ion mass tolerance of 0.60 Da and a parent ion tolerance of 10.0 PPM. Carbamidomethyl of cysteine was specified as a fixed modification. Variable modifications were specified including methionine oxidation and acetylation of peptide n-termini. For protein identification and quantification, a cut-off value of at least one unique high confidence peptide per protein, corresponding to a 1% false discovery rate (FDR) at the peptide level and peptide rank 1 protein was selected. Proteins and peptides identified and quantified by at least two of three replicates were used for comparisons. Mass spectrometry data were obtained at the University of Massachusetts Amherst Mass Spectrometry Center. Proteome data is deposited in the EMBL-EBI PRoteomics IDEntifcations Database (PRIDE) under Accession: PXD021868.

### Proteome Data Analysis

Scaffold version 4.9.0, (Proteome Software Inc., USA) was used to validate peptide and protein calls. Peptide identifications were accepted at >99.0% probability by the Peptide Prophet algorithm (28) with Scaffold delta-mass correction. Protein identifications were accepted at >99.0% probability and containing at least two identified peptide. Protein probabilities were assigned by the Protein Prophet algorithm (29). Proteins that contained similar peptides and could not be differentiated based on MS/MS analysis alone were grouped to satisfy the principles of parsimony. In order to generate relative quantification across samples, result files from Proteome Discoverer were imported into Scaffold and sf3 files were exported for the qualitative and quantitative comparisons. Peptide and protein abundances were normalized based on total sum of peptide and protein abundances, respectively, and were log10 transformed prior to the analysis. Multivariate data analysis was carried out using MetaboAnalyst 4.0 (30) (www.metaboanalyst.ca). Partial Least Squares Discriminant Analysis (PLS-DA) was used to maximize the covariance between variables. Variable importance of projection (VIP) score plot obtained by PLS-DA identified proteins (VIP score of ≥1.0) responsible for the clustering of groups. Proteins were annotated with GO terms using UniProt ID mapping (www.uniprot.org) and KEGG metabolic pathways were reconstructed using KEGG Mapper. PLS-DA, VIP, and KEGG metabolic pathways are reported in Supplementary Figures 1–3. The obtained proteomes were queried for proteins involved in the hypothetical *B. infantis* fucose metabolic pathway based on putative fucose genes identified in the *B. infantis* genome.

### RNA-seq Transcriptome Library Preparation

For RNA extraction, cells stored in RNAlater (MilliporeSigma, Burlington, MA) at −80°C were centrifuged at 14,000 × g for 3 min and cell pellets were washed with 1 mL cold 1X PBS buffer. Total RNA was extracted using Ambion RNAqeous kit (Life Technologies, USA) according to manufacturer instructions with a beadbeating step added to disrupt the cells. Accordingly, cells were suspended in 600 μL lysis buffer in beadbeating tubes (100 μm Silica Beads; OPS Diagnostics, USA) at 5.5 m/s for 30 sec 1X using a FastPrep 24 bead beader (MP Biomedicals, USA). RNA concentrations were measured by a NanoDrop spectrophotometer and samples were subjected to DNase treatment using the Ambion Turbo DNA-free kit (Invitrogen, USA) using 2 μL of DNase I for 45 min for 25 μL eluted total RNA. The RNA was evaluated for genomic DNA contamination using qRT-PCR performed on a 7500 Fast Real-Time PCR System (Applied Biosystems, USA) as described previously (31). An additional round of DNase treatment was performed for samples with high levels of residual DNA.

DNase-treated RNA was quantified using the Qubit High Sensitivity RNA Assay Kit (Life Technologies, USA) and RNA quality was assessed through RNA integrity number equivalent (RIN^e^) as determined on a TapeStation 2200 system using High Sensitivity RNA screen tapes and reagents (Agilent Technologies, USA). Samples with RIN^e^ >7.0 were subjected to ribosomal RNA depletion and mRNA purification with the RiboMinus kit with Magnetic Bead Clean up Module using the custom Pan-Prokaryote Probe Mix to target bacterial rRNA (Thermo Fisher Scientific, USA). RNA was quantified using the Qubit High Sensitivity RNA Assay and rRNA depletion was confirmed on TapeStation 2200 system. The depleted RNAs (RIN^e^ ~1.0–2.5) proceeded to sequencing library generation with the NEBNext Ultra II Directional Library preparation kit (New England Biolabs, USA). Purification was performed with AmPure XP beads (Beckman Coulter, USA) and indexed using NEBNext® Multiplex Oligos for Illumina Indices (Dual Index Primers Set 1; New England Biolabs Inc., USA) with 8–10 PCR cycles which varied according to input RNA concentration. The libraries were quantified using the Qubit double-stranded DNA (dsDNA) HS assay (Life Technologies, USA). The quality of library products was measured by high sensitivity DNA screen tapes on the TapeStation 2200 system. Sequencing libraries were pooled in equimolar concentrations (4 nM) and denatured immediately prior to sequencing following Illumina pooled library instructions. Sequencing was performed on an Illumina NextSeq platform (NextSeq 500/550 High Output Kit v2.5, paired-end, 150 cycles, 5% Phi-X) at the Genomics Resource Laboratory, University of Massachusetts Amherst. Raw reads are publicly deposited in the NCBI Gene Expression Omnibus database (https://www.ncbi.nlm.nih.gov/geo/) under accession number GSE159189.

### Transcriptome Informatic and Statistical Analysis

Raw reads were uploaded to the Massachusetts Green High-Performance Computing Cluster used for all computational/statistical analyses unless specifically noted. Sequencing adaptors were trimmed during de-multiplexing. The reads were aligned to the reference *Bifidobacterium longum* subsp. *infantis* ATCC 15697 (NCBI accession number: NC 011593.1) using bowtie-2 (v2.3.4.3) (32). Total and non-unique gene reads aligning to a specific genomic locus, as well as calculated raw counts (HTSeq count, v0.10.0) (33) were obtained for differential expression analysis.

### Differential Gene Expression

In order to identify and quantify the magnitude of differentially expressed genes, the R package DESeq2 was used to analyze the raw count data (34). Genes with a sum count <50 were removed from analysis by pre-filtering. DESeq2 applies the Wald test for statistical analysis with adjusted *p* ≤ 0.05 designated as statistically significant. Principal component analysis (PCA) was performed and plots were visualized using DESeq2 (34). Gene maps were created and visualized using the R package genoplotR following *B. infantis* ATCC 15697 reference genome obtained from NCBI (NC_011593.1) and colored with corresponding z–score (35, 36). Z–score for each gene of interest was calculated using the following equation:

$$\begin{array}{l} \text{z}-\text{score}=\frac{Expression-Mean\;expression\;across\;all\;samples}{Standard\;deviation} \end{array}$$

where expression corresponds to the normalized counts of the gene of interest. Z-scores were visualized using a heatmap or gene map. Volcano plots were created and visualized using the R package EnhancedVolcano (37). Predicted gene functions were determined using a combination of BLAST (38) and KEGG (http://www.genome.jp/kegg~searches).

### Statistical Analyses

Statistical analysis of growth kinetics was performed using the final OD_600_ as a measure of total biomass. Final OD_600_ was subjected to one-way analysis of variance and Tukey's HSD for multiple comparisons between carbohydrate source. Metabolite concentrations were subjected to one-way analysis of variance and Tukey's HSD test for multiple comparisons between carbohydrate source. Fold change in gene expression was subjected to the Wald test during processing in DESeq2 (34). Z-scores generated from normalized counts were subjected to one-way analysis of variance and Tukey's HSD test for multiple comparisons between 2′FL concentrations and carbohydrate source.

### Experimental Layout

Co-fermentation of free fucose with glucose and lactose in both large volume (5 mL) and standard 96-well microplate formats was performed. Large volume cultures were used to obtain sufficient biomass to perform intra- and extracellular metabolite assays as a function of growth time during co-fermentation of free fucose and glucose as well as during growth on 2′FL as a sole carbohydrate. Proteomic and transcriptomic analyses were performed using large volume cultures in order to obtain sufficient biomass to extract the necessary quantities of protein and RNA. 96-well microplate growth assays were performed in all other cases where growth kinetics and metabolite production were examined. A brief schematic of the experimental layout is included in Supplementary Figure 4.

## Results and Discussion

### *B. infantis* Utilizes Free Fucose as a Carbohydrate Source

**Figure 1:**
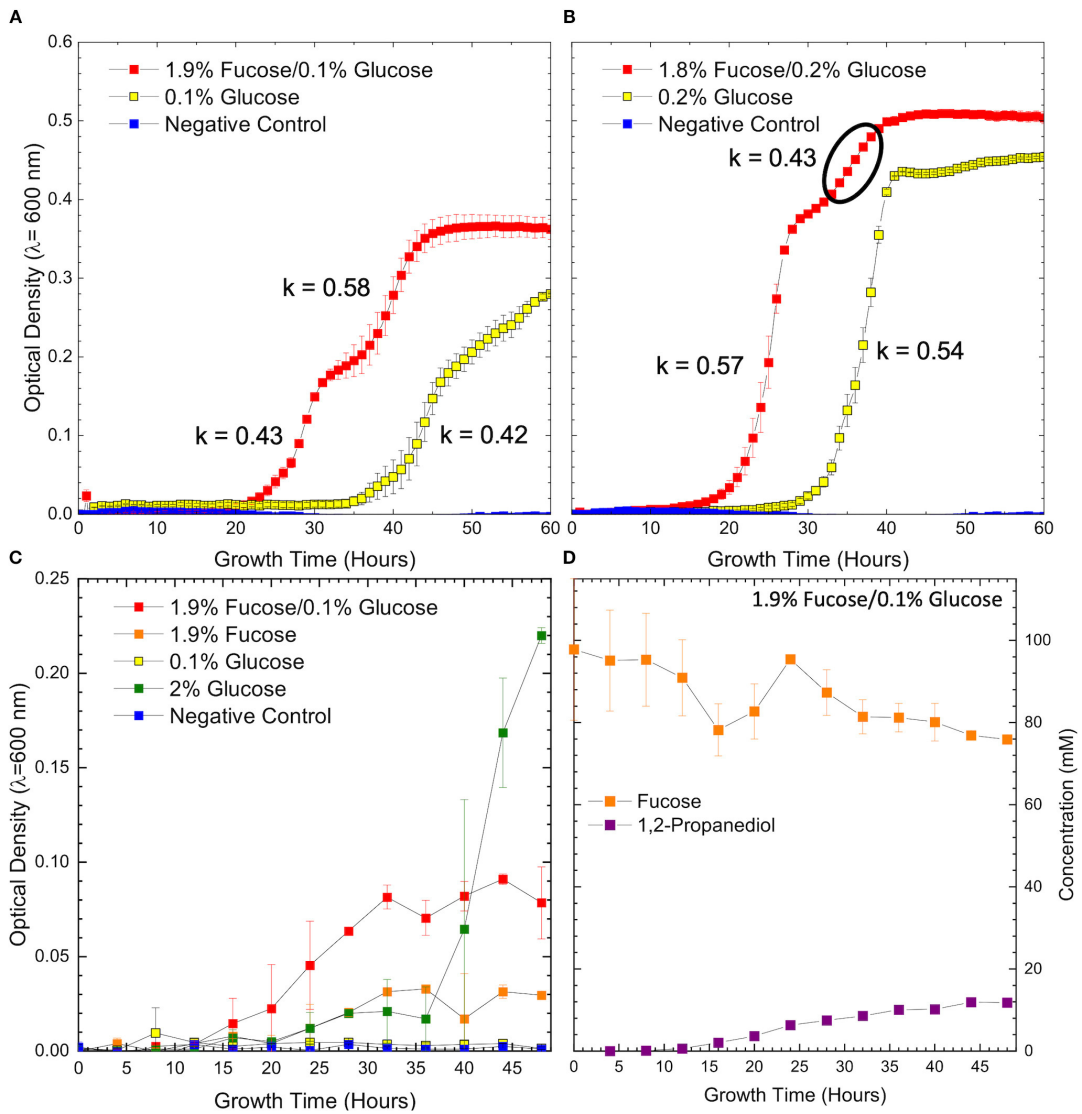
Growth curves of ATCC 15697 in **(A)** co-fermentation of 1.9% fucose and 0.1% glucose, **(B)** co-fermentation of 1.8% fucose and 0.2% glucose and on a corresponding concentration of glucose as a sole carbohydrate source shows distinct changes in growth rate during co-fermentation. The region where growth kinetics deviate and are best fit with a second growth rate is noted by the bold circle. Growth curves of ATCC 15697 on **(C)** co-fermentation of 1.9% fucose and 0.1% glucose and on individual carbohydrate sources. Samples were analyzed for metabolite concentrations at each data point. Concentrations of fucose and 1,2-propanediol **(D)** during co-fermentation of 1.9% fucose and 0.1% glucose are inversely related and in stoichiometric agreement with the proposed fucose metabolic pathway. Error bars denote standard error of the mean.

In order to understand *B. infantis* metabolism of free fucose and HMO-derived fucose, *B. infantis* ATCC 15697 was subjected to growth on fucose as a sole carbohydrate source and in co-fermentation with other sugars. Co-fermentation was performed due to prior observation of inconsistent *B. infantis* growth on fucose as the sole carbohydrate source which increased with the addition of trace glucose (14, 20, 21). Growth was performed in a standard 96-well plate format to maximize sensitivity and throughput and percentage carbohydrate substrate added are reported as wt/vol. Consistent with Bunesova et al. (14) supplementing free fucose with a trace amount of glucose (0.1–0.2%) resulted in higher final biomass (Figures 1A,B). *B. infantis* ATCC 15697 growth on free fucose as a sole carbohydrate source was reproducible and consistent across biological replicates. Co-fermentation of 0.1% glucose and 1.9% fucose resulted in significantly greater growth (final OD_600_ = 0.38 ± 0.09) relative to 0.1% glucose alone (final OD_600_ = 0.22 ± 0.08, *p* = 9.9E-5) (Figure 1A). Moreover, growth on 1.8% fucose and 0.2% glucose (final OD_600_ = 0.50 ± 0.06) is also significantly higher than growth on 0.2% glucose alone (final OD_600_ = 0.44 ± 0.002, *p* = 0.02) (Figure 1B). Growth on 1.8% fucose and 0.2% glucose is also significantly higher than growth on 1.9% fucose and 0.1% glucose (*p* = 0.003). Growth on 0.2% glucose is significantly higher than growth on 0.1% glucose (*p* = 2.04E-8).

Growth in co-fermentation at both concentrations of fucose and glucose exhibits two distinct regions with different growth rates. In both conditions, the growth rate in the first regime (k = 0.43, k = 0.57) is in concordance with the growth rate observed in fermentation of glucose as a sole carbohydrate source (k = 0.42, k = 0.54). Given the shift in growth rate and the relative length of each region, it is possible that the cells are transitioning from glucose to fucose metabolism as the limiting concentration of glucose is exhausted. Depletion of the preferred carbohydrate source is the most common driver of diauxic shifts in microbial systems. Interestingly, the growth rate increases across this diauxic transition during co-fermentation of 1.9% fucose and 0.1% glucose (k = 0.43–0.58) and decreases during co-fermentation of 1.8% fucose and 0.2% glucose (k = 0.57–0.43). Thus, it is possible that the initial concentration of glucose influences the direction of growth rate change. Biomass accumulation on 0.1% glucose is severely restricted indicating that *B. infantis* ATCC 15697 does not have sufficient substrate at this concentration. Therefore, it is concluded that increased growth in co-fermentation is due to the inclusion of fucose as a fermentable carbohydrate.

In order to assess metabolite production as a function of growth, larger volume co-fermentation was performed to identify intracellular metabolites from sufficient biomass. Higher volume co-fermentation of glucose and fucose (Figure 1C) was consistent with the smaller volume 96-well format (Figures 1A,B), although exhibited lower OD_600_ values overall. Again, growth in co-fermentation of 0.1% glucose and 1.9% fucose was significantly increased relative to 0.1% glucose (*p* = 0.004) and 1.9% fucose (*p* = 0.02) as a sole carbohydrate source. In the large volume format, *B. infantis* ATCC 15697 does not significantly utilize free fucose as a sole carbohydrate source relative to the negative control (i.e., no added sugar) (*p* = 0.1) (Figure 1C). These results are consistent with previous observations that several *B. infantis* strains do not grow efficiently on fucose and only grow in the presence of trace glucose (14).

In contrast, co-fermentation of 0.1% lactose and 1.9% fucose is driven predominately by lactose metabolism as suggested by insignificant difference to final OD_600_ relative to growth on 0.1% lactose alone (Supplementary Figure 5, *p* = 0.07). This is not surprising given the efficient metabolism of lactose by *B. infantis* ATCC 15697 which likely prioritizes lactose over fucose. The presence of a second growth phase in co-fermentation of lactose and fucose (Supplementary Figure 5) suggests that fucose is being metabolized, similar to co-fermentation of the same concentrations of glucose and fucose (Figure 1A). Fucose metabolism during co-fermentation with lactose is supported by 1,2-PD production and disappearance of fucose from the medium (Figure 1D, Supplementary Table 1).

### 1,2-Propanediol Is Produced in Stoichiometric Accordance With Free Fucose Utilization

Given that *B. infantis* ATCC 15697 co-ferments limiting concentrations of glucose or lactose and excess fucose, as well as free fucose, fermentative intermediates were profiled to clarify the fucose utilization pathway. A mechanistic pathway (Supplementary Figure 6) was proposed by Bunesova *et al* (14) and supported by James *et al* in *Bifidobacterium kashiwanohense* and *Bifidobacterium breve* (11). This hypothetical pathway reflects rhamnose metabolism in *Campylobacter* sp. and *X. campestris* and features several intermediate metabolites to be potentially assayed (11, 14). L-fucono-1,4-lactone was selected as a metabolite marker to track due to its stability, exclusivity to the putative fucose pathway, and it yields a single spectral peak. Conversely, L-fuconate exhibits several peaks that are interspersed among the linear form of fucose. As such, intracellular metabolites were measured during co-fermentation of 0.2% glucose and 1.8% fucose. The higher concentration of glucose was used in order to achieve sufficient biomass to reliably detect L-fucono-1,4-lactone that is expected to be present in low concentrations. The presence of a lactate, pyruvate, and 1,2-PD signal confirmed that sufficient biomass was generated to detect intracellular metabolites (data not shown).

Despite the predicted stability of L-fucono-1,4-lactone, the metabolite was not detected at any point during growth on 0.2% glucose and 1.8% fucose (Supplementary Figure 7). There is, however, evidence of intracellular 1,2-PD as it was observed in the GC-MS chromatogram as a shallow, broad peak appearing very close to the solvent (data not shown). If fucose is metabolized through a L-fucono-1,4-lactone intermediate it may be highly transient or present in concentrations below the limit of detection. Although intracellular L-fucono-1,4-lactone was not detected, the presence of intracellular 1,2-PD indicates that fucose metabolism proceeds during biomass accumulation. Given that 1,2-PD is a secreted metabolic end product and expected to be found in higher concentrations in the extracellular matrix relative to intracellular levels, extracellular 1,2-PD served as a marker for fucose metabolism. Accordingly, we hypothesized that *B. infantis* ATCC 15697 secretes extracellular 1,2-PD corresponding with a decrease in fucose as a hallmark of fucose metabolism (Figure 1D).

Unlike glucose or galactose, fucose is not predicted to enter the bifidobacterial central metabolic pathway, termed the bifid shunt. This assumption is based on fucose metabolism in other bacterial systems and on the unique characteristics of the bifid shunt. Instead, it is hypothesized that an equimolar ratio of pyruvate and 1,2-PD is produced per mole of fucose consumed. Thus, 1,2-PD would be produced at the same rate that fucose is expended. Pyruvate would be catabolized and secreted as lactate, formate, and/or acetate to recycle cofactors. Extracellular metabolites produced during growth on fucose are provided in Table 1. 1,2-PD is produced at 0.5 times fucose consumption during co-fermentation of fucose and glucose. During growth on fucose as a sole carbohydrate source, 1,2-PD is produced at 0.44 times the consumption of fucose. While these ratios are not the expected equimolar production, it is important to note that the uncertainty in fucose concentration is large. Accounting for uncertainty in fucose concentration provides a range of the ratio of 1,2-PD production to fucose disappearance of 0.3–1.4 during co-fermentation of fucose and glucose and 0.3–0.7 during growth on free fucose. Furthermore, some deviation from the theoretical ratio of 1 is anticipated due to variation in growth state. Overall, *B. infantis* ATCC 15697 is observed to produce 1,2-PD as a result of fucose metabolism.

**Table 1:** *B. infantis* extracellular metabolite production during growth in co-fermentation of fucose and glucose and on fucose and glucose as sole carbohydrate sources.

	**Glucose (mM)**	**Fucose (mM)**	**Lactate (mM)**	**Formate (mM)**	**Acetate (mM)**	**1,2-PD (mM)**	**Ace:Lac**	**Form:Ace**
1.9% fucose/0.1% glucose	−5 ± 1	−26 ± 16	+1.6 ± 0.6	+11.8 ± 0.6	+15.0 ± 0.1	+13 ± 1	3.08	0.70
1.9% fucose	ND	−20 ± 7	+0.3 ± 0.8	+6.7 ± 0.9	+8.0 ± 0.7	+8.8 ± 0.2	2.26	1.02
0.1% glucose	−0.96 ± 0.09	ND	+0.10 ± 0.07	+0.1 ± 0.1	+0.58 ± 0.1	ND	0.71	0.99

*Change in concentration is calculated using extracellular concentrations prior to growth and following stationary phase. ND, not detected*.

The acetate:lactate ratio during co-fermentation of glucose and fucose surpasses the theoretical ratio of 1.5 secreted during hexose metabolism (Table 1). As compared to efficiently fermented carbohydrates such as lactose (Table 2), lactate concentrations greatly decreased and formate concentration increased during growth on fucose. This metabolic shift to favor formate secretion may be to prioritize ATP production through a branch in the bifid shunt. Similar observations have been reported for *B. infantis* ATCC 15697 (39) and other *Bifidobacterium* species (40) during inefficient metabolism of neutral HMOs. An acetate:lactate ratio of 0.99 during growth on 0.1% glucose may be due to poor growth on such a limiting sugar concentration, and thus deviates from the theoretical ratio of 1.5.

**Table 2:** *B. infantis* extracellular metabolite production during growth on 2′fucosyllactose is similar to that both in co-fermentation of fucose and lactose and on fucose and lactose as sole carbohydrates.

**Carbohydrate**	**Lactate (mM)**		**Acetate (mM)**		**Formate (mM)**		**1,2-PD (mM)**		**Δ[Fucose] (mM)**	**Ace:Lac**	**Form:Ace**
	**Raw**	**Norm**	**Raw**	**Norm**	**Raw**	**Norm**	**Raw**	**Norm**			
0.8% 2′FL	16 ± 1	16 ± 1	40.0 ± 0.8	40.0 ± 0.8	11.9 ± 0.5	11.9 ± 0.5	7.6 ± 0.1	7.6 ± 0.1	−7.5 ± 0.4	2.5	0.3
0.8% fucose	3.14 ± 0.08	18.97 ± 0.08	3.5 ± 0.2	21.3 ± 0.2	2.6 ± 0.5	15.6 ± 0.5	5.30 ± 0.08	21.01 ± 0.08	−5.3 ± 0.9	1.1	0.7
0.8% lactose	30.2 ± 0.5	22.9 ± 0.5	56 ± 1	42 ± 1	3.6 ± 0.2	2.7 ± 0.2	ND	ND	ND	1.8	0.06
0.8% fucose/0.8% lactose	28.1 ± 0.3	22.4 ± 0.3	45.5 ± 0.7	36.3 ± 0.7	3.9 ± 0.3	3.1 ± 0.3	4.07 ± 0.05	3.25 ± 0.05	−4.8 ± 0.4	1.6	0.09

*Change in concentration is calculated using extracellular concentrations prior to growth and following stationary phase. ND, not detected*.

Increased formate:acetate ratios are consistent with a shift in pyruvate metabolism. When more than 50% acetyl-CoA is converted to acetate by *B. longum*, the theoretical formate:acetate ratio is approximately 0.4 (41). Here, *B. infantis* ATCC 15697 has surpassed that threshold, suggesting that there is a shift to produce more acetyl-CoA than lactate from pyruvate. The increased ratios are expected with the inefficient fermentation of fucose as a sole carbohydrate source. These results support the hypothesis that *B. infantis* ATCC 15697 utilizes free fucose as a carbohydrate to support growth, although fucose fermentation is somewhat inefficient under these experimental conditions.

### 1,2-Propanediol Is Produced in Stoichiometric Accordance With 2′FL Utilization

**Figure 2:**
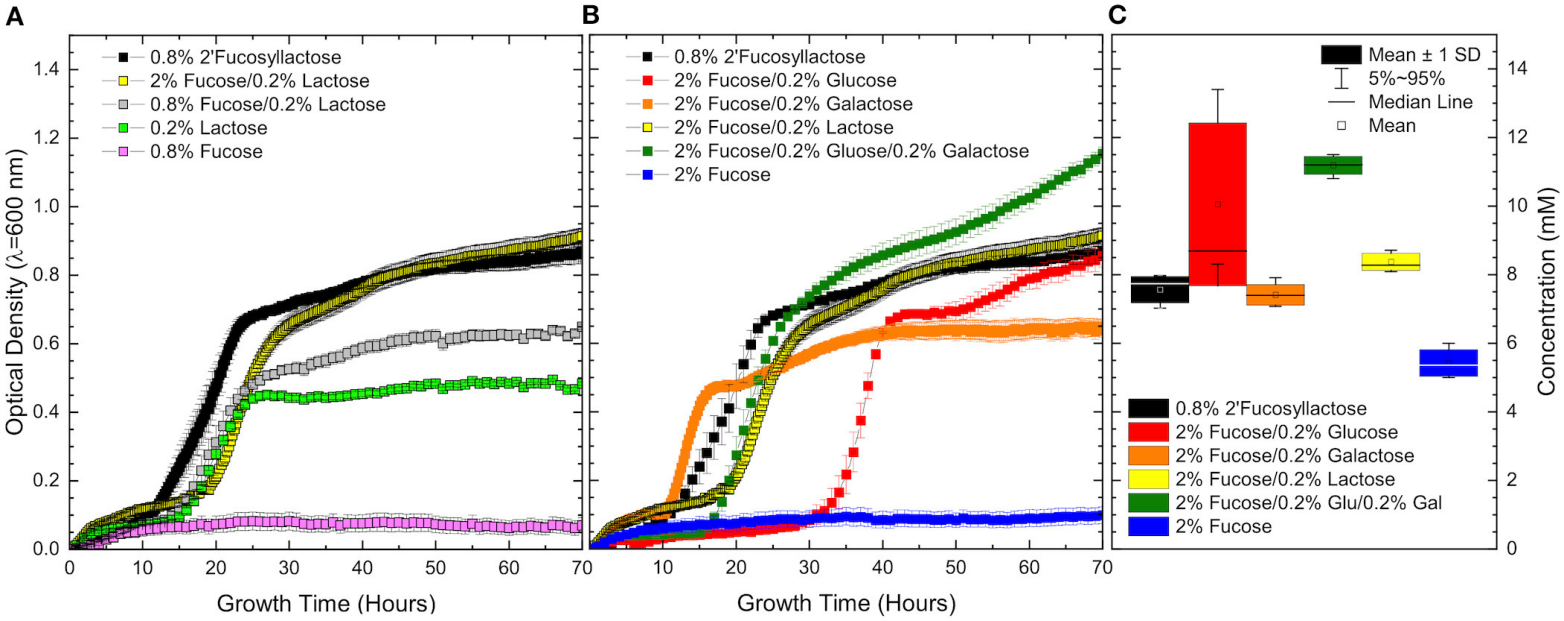
Growth curves of ATCC 15697 growth in **(A)** co-fermentation of excess fucose and limiting lactose as compared to 2′fucosyllactose. Co-fermentation of fucose and limiting lactose shows similar behavior to fermentation of 2′fucosyllactose and results in increased growth over individual carbohydrate sources. Co-fermentation of **(B)** excess fucose and limiting concentrations of mono- and di-saccharide components of 2′fucosyllactose shows a consistent synergistic effect. 1,2-Propanediol production **(C)** during co-fermentation is equal to or higher than 2′fucosyllactose and higher than growth on fucose as a sole carbohydrate source. Error bars denote standard error of the mean.

Similar to fucose metabolism, 2′FL is metabolized by *B. infantis* ATCC 15697 to produce 1,2-PD (Figures 2A–C) when fermenting substrates at slightly higher concentrations than those found in human milk (42). Each molecule of 2′FL is composed of a lactosyl and fucosyl moiety, thus utilization of 2′FL approximates fucose utilization. As with co-fermentation of fucose and glucose, 1,2-PD production would be expected to be equivalent to fucose. In three tested conditions (0.8% 2′FL, 0.8% fucose, and 0.8% fucose/0.8% lactose) the decrease in fucose or 2′FL concentration is nearly identical to the production of 1,2-PD (Table 2). In both instances formate production is increased fivefold (5.8-fold for free fucose and 4.4-fold for 2′FL) in a comparison of free fucose and 2′FL metabolism (Table 2). Moreover, lactate production is decreased relative to growth on lactose when normalized to final OD_600_. In contrast, acetate production is reduced while subsisting on fucose which could be an artifact of poor growth or otherwise confounded due to metabolism of the lactosyl component of 2′FL. A co-fermentation of 0.8% fucose and 0.8% lactose was used to functionally approximate fermentation of integrated 0.8% 2′FL as a sole carbohydrate source (Table 2). Whereas, absolute metabolite concentrations of the fucose/lactose co-fermentation are in closer agreement with those during growth on 2′FL, the formate and 1,2-PD concentrations during fucose metabolism are reduced with the addition of the more efficiently utilized lactose. As such, it is difficult to derive conclusions regarding free fucose metabolism in direct comparison to 2′FL-derived fucose.

### Limiting Concentrations of Mono- and Disaccharide Residues of 2′FL Increase Fucose Metabolism

In order to clarify the boundaries of fucose metabolism in the presence of a preferred sugar, *B. infantis* was grown in co-fermentation of excess fucose and limiting concentrations of lactose, glucose, and galactose. Initial co-fermentation of limiting lactose (0.1%) (Supplementary Figure 5) resulted in variable growth indicating that this concentration is too low to consistently support growth. Thus, co-fermentation of 2% fucose and slightly higher concentrations (0.2%) of glucose, galactose, and lactose were used (Figure 2B). Co-fermentation of fucose, glucose, and galactose produced the highest biomass as measured by final OD_600_ (Figure 2B). Co-fermentation of fucose with glucose or lactose produced roughly equivalent final OD_600_ as fermentation of 2′FL as the sole carbon source. In contrast, co-fermentation of fucose and galactose produced a lower final OD_600_. Regardless of the co-fermented substrate, growth in co-fermentation with fucose achieved higher biomass than growth on fucose or the limiting carbohydrate alone. Furthermore, the final OD_600_ achieved in co-fermentation was higher than the sum of final OD_600_ achieved during growth on individual components as sole carbohydrate sources (lactose shown in Figure 2A, additional sugars not shown).

In accordance with increased growth, final 1,2-PD concentrations increased during growth in co-fermentation relative to growth on 2% fucose as a sole carbohydrate source (Figure 2C). All co-fermentations exhibit significantly higher 1,2-PD secretion relative to growth on fucose alone [fucose/glucose (*p* = 6.76E-8), fucose/galactose (*p* = 0.02), fucose/lactose (*p* = 1.24E-4), fucose/glucose/galactose (*p* = 1.79E-8)]. Moreover, 1,2-PD production was unchanged (galactose or lactose) or increased [glucose (*p* = 0.003), glucose/galactose (*p* = 1.65E-7)] during co-fermentation as compared to 1,2-PD production during growth on 0.8% 2′FL. Fucose metabolism and central carbohydrate metabolism are linked as evident from increased final OD_600_ corresponding to increased 1,2-PD concentration.

Clearly fucose is not a preferred carbohydrate source for *B. infantis* ATCC 15697 and is not efficiently metabolized as a sole carbohydrate source regardless of the addition of limiting co-fermented sugars. Despite this inefficient metabolism, co-fermentation significantly increases growth and hallmarks of fucose metabolism. This phenomenon was also observed by Bunesova *et al* in that bifidobacterial growth on free fucose as a sole carbohydrate source was greatly improved by the inclusion of trace (0.4 mM) glucose (14). It is possible that a metabolite represses the fucose pathway as a result of fucose catabolism or carbon flux through the bifid shunt. James et al. putatively linked a LacI-family regulator in *B. kashowanihense* (*fumR)* to fucose metabolism, although mechanistic details of regulatory interactions remain unresolved (11). *B. infantis* ATCC 15697 possesses a homolog of *fumR* (Blon_RS01775) and this putative repressor may restrict fucose metabolism in the presence of sufficient concentrations of efficiently utilized sugars (e.g. glucose, galactose, and lactose). It is possible that fucose transport is repressed in the presence of high concentrations of efficient substrates, similar to the preferential metabolism of lactose over glucose observed in *B. longum* (43). In this experimental system, fucose transport is likely not hindered by excess lactose, as extracellular fucose concentrations diminish uniformly regardless of growth solely on fucose or in co-fermentation with lactose (Table 2). The variability within LacI-family allosteric activities suggest that bifid shunt and/or fucose-derived metabolites potentially regulate fucose metabolism (44). Furthermore, fucose metabolism is not repressed by limiting co-fermented carbohydrates. This is evidenced by similar concentrations of secreted 1,2-PD on excess 2′FL and excess fucose co-fermented with limiting sugars.

### Utilization of Higher Concentrations of 2′FL Does Not Promote Efficient *B. infantis* Growth

**Figure 3:**
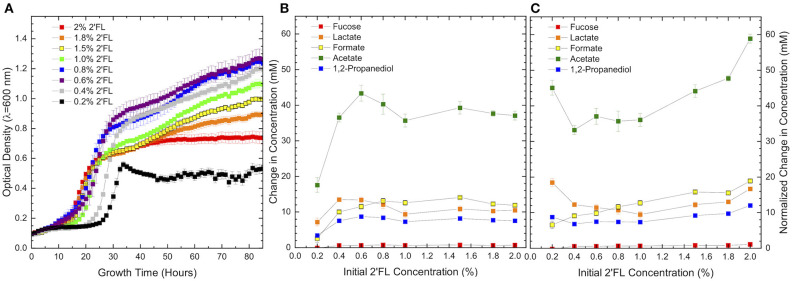
Growth curves of ATCC 15697 growth on **(A)** 2′fucosyllactose as a sole carbohydrate source is highly concentration-dependent. Metabolite production **(B)** appears to be consistently dependent on 2′fucosyllactose concentration, but concentration dependence does not persist when **(C)** metabolite concentrations are normalized to account for differences in biomass. Error bars denote standard error of the mean.

2′FL concentrations in human milk vary between and within populations depending on multiple factors including maternal secretor status (6, 7, 42, 45–47). In order to test the hypothesis that the fucose utilization phenotype responds to 2′FL in a concentration-dependent manner, *B. infantis* ATCC 15697 was grown on 2′FL as the sole carbohydrate source at concentrations ranging from 0.2% (i.e., limiting) to 2% (i.e., exceeds human milk concentrations). Thus, concentration-dependent growth on 2′FL would result in higher final OD_600_ and higher 1,2-propanediol production. Interestingly, and counter to this hypothesis, 2% 2′FL promotes moderate *B. infantis* ATCC 15697 growth, reaching an OD_600_ of 0.64 ± 0.06. Accumulated biomass increases with diminishing 2′FL concentrations (Figure 3A) until 2′FL is limiting to exhibit an inverse relationship. A more pronounced diauxic curve is exhibited as 2′FL concentration falls below 1% and is suppressed again at concentrations below 0.6%. It is currently unclear how this concentration-dependence relates to fucose metabolism.

Absolute concentrations of secreted metabolites follow the parabolic trend observed in final OD_600_ (Figure 3B); however, this trend does not hold when metabolite concentrations are normalized to final OD_600_ to account for biomass variation (Figure 3C). As a result, the discordance between cellular growth and metabolite production is apparent and indicates a significant biological phenomenon. When normalized to biomass, formate is secreted in a consistently positive slope with increasing 2′FL. Acetate production also exhibits a net positive slope except at the lowest 2′FL concentration, which is severely limiting. The increase in formate and acetate production suggest that metabolic flux increases along with 2′FL concentrations. Formate production is small relative to the increase in acetate concentrations coinciding with increasing 2′FL concentration and, thus, does not significantly change the formate:acetate ratio (Table 3). Whereas normalized formate and acetate production are concentration-dependent, normalized lactate production does not exbibit a consistent relationship with 2′FL concentration. The relatively constant lactate concentration is consistent with increased formate and acetate production given that these metabolites are often increased at the expense of lactate production (41, 48–51). The specific metabolic end products that were quantified were not significantly impacted by 2′FL concentration despite significant changes in biomass accumulation with changing 2′FL concentration.

**Table 3:** *B. infantis* 2′fucosyllactose consumption and 1,2-propandiol production during growth on 2′fucosyllactose as a sole carbohydrate source is not concentration-dependent when differences in final biomass are accounted for. However, acetate:lactate and formate:acetate ratios show some subtle variation with 2′fucosyllactose concentration.

**2^**′**^FL conc. (%w/v)**	**2%**	**1.8%**	**1.5%**	**1%**	**0.8%**	**0.6%**	**0.4%**	**0.2%**
Final OD_600_ (± stdev	0.63 ± 0.04	0.79 ± 0.02	0.89 ± 0.01	0.99 ± 0.02	1.13 ± 0.01	1.17 ± 0.06	1.10 ± 0.02	0.39 ± 0.03
Acetate:Lactate	3.2	3.2	3.3	3.3	2.9	2.9	2.4	2.0
Formate:Acetate	0.34	0.34	0.37	0.37	0.34	0.29	0.31	0.24
Δ[2′FL] (mM)	−7 ± 2	−5 ± 2	−2 ± 1	−6 ± 2	−7 ± 1	−8 ± 2	−11.1 ± 0.3	−7 ± 2
Δ[1,2–PD] (mM)	+7.57 ± 0.07	+7.7 ± 0.1	+8.2 ± 0.2	+7.29 ± 0.05	+8.4 ± 0.4	+8.7 ± 0.4	+7.5 ± 0.1	+3.4 ± 0.2
Normalized Δ[2′FL] (mM)	−14 ± 2	−7 ± 2	−2 ± 1	−6 ± 2	−6 ± 1	−7 ± 2	−10.1 ± 0.3	−17 ± 2
Normalized Δ[1,2-PD] (mM)	+12.02 ± 0.07	+9.8 ± 0.1	+9.2 ± 0.2	+7.36 ± 0.05	+7.4 ± 0.4	+7.5 ± 0.4	+6.8 ± 0.1	+8.8 ± 0.2

*Change in extracellular concentration is calculated using concentrations prior to growth and following stationary phase. ND, not detected*.

A potential factor in inhibition of growth with increasing 2′FL concentration is a potential toxicity of 1,2-PD, as 1,2-PD concentration increases with that of 2′FL (Figure 3B). To test this, a growth inhibition assay indicated that 1,2-PD is only slightly inhibitory between 168 and 0.08 mM with a difference in OD_600_ of 0.107 ± 0.007 (*p* = 9.5E-8) between the highest and lowest 1,2-PD concentrations. Similarly, the growth inhibition assay of lactate (i.e., inhibitory at high concentrations) indicates a similar slight inhibition between 144 and 0.07 mM lactate (ΔOD_600_ of 0.150 ± 0.005, *p* = 3.0E-7). Neither metabolite accumulates to inhibitory concentrations prior or during growth. 1,2-PD reaches a maximum concentration of 8.6 ± 0.4 mM at 0.6% 2′FL. Therefore, it is unlikely that secreted 1,2-PD or other quantified end-product metabolites impact growth at higher concentrations of 2′FL. It is possible that fucose metabolism is repressed by some unknown mechanism. Thus, proteomics and transcriptomics were conducted to examine global regulatory networks during fucose metabolism to better understand the relationship between fucose, lactose, and 2′FL utilization.

### The Proteome Contains Hallmarks of Putative Bifidobacterial Fucose Metabolic Pathway

**Figure 4:**
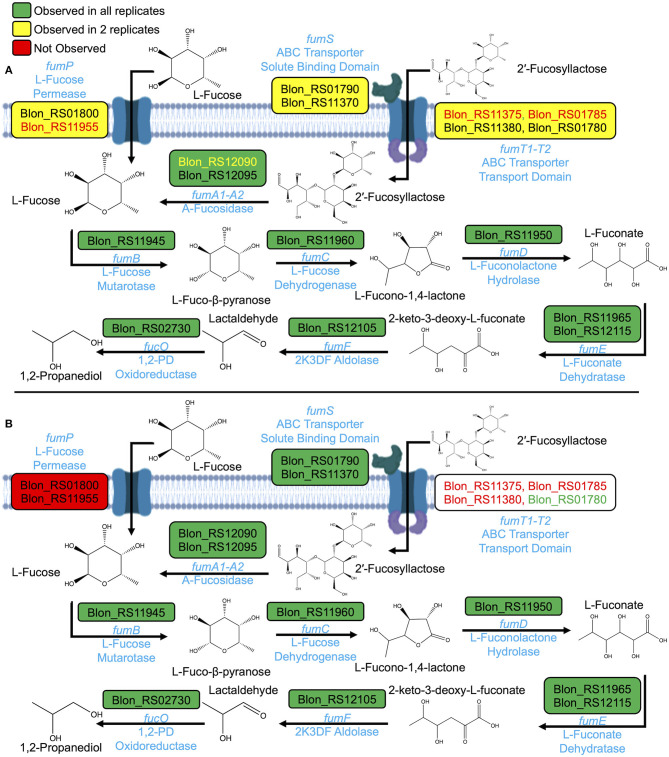
Proposed fucose pathways based on proteome during growth on **(A)** free fucose and **(B)** 2% 2′fucosyllactose as sole carbohydrate sources. Gene symbols are given in light blue above the reaction arrows and corresponding protein names below.

The *B. infantis* proteome was interrogated for signals of pathways active during fucose catabolism. The proteome during growth on free fucose as a sole carbohydrate exhibits features consistent with the pathway proposed for *B. kashowanohense*, albeit yet to be validated incisively (Figure 4A) (11, 14) Figure 4 integrates proteins within the proteome encoded by the genes of interest in the hypothetical *B. infantis* fucose metabolic pathway. Putative fucose metabolism proteins were observed in at least two of three biological replicates. Transport-associated proteins were identified in relatively low abundance, which may be due to their spanning the cell membrane and, thus, tendency to collapse in the insoluble proteome fraction. Nevertheless, transport proteins were observed in two of three biological replicates.

An ABC transporter linked to 2′FL (Blon_RS01780-RS01790) (4) is expressed during growth on fucose as a sole carbohydrate. This indicates that fucose signals 2′FL metabolism to initiate and reflects likely overlapping processes to catabolize free or integrated fucose. Partial evidence of a secondary transporter (Blon_RS11370-RS11380) was identified in the proteome. Proteins corresponding to Blon_RS11370 (FumS) and Blon_RS11380 (FumT2) were detected in two of three replicates, and Blon_RS11375 (FumT1) was not observed.

The fucose proteome is in agreement with 1,2-PD production (Table 1). Although intracellular L-fucono-1,4-lactone was not detected *via* GC-MS, proteins with KEGG orthologs corresponding to L-fucono-1,4-lactone and L-fuconate synthesis were present in the proteome. These proteins with putative fucose metabolic functions include L-fucose dehydrogenase (FumC), L-fuconolactone hydrolase (FumD), as well as L-fuconate dehydratase (FumE). Furthermore, differential expression of L-fuconolactone and L-fuconate-related proteins was observed during growth on free fucose as compared to growth on lactose. FumD (Blon_RS11950) showed a log_2_ fold change of 13.1 (*p* = 5.95E-7) and two orthologs of FumE (Blon_RS11965 and Blon_RS12115) showed log_2_ fold changes of 15.4 (*p* = 1.39E-5) and 1.7 (*p* = 0.02). If these enzymes function as predicted, L-fucono-1,4-lactone and L-fuconate may be produced as highly transient intermediates during fucose metabolism.

### 2′FL Induces a Proteomic Signature That Reflects a Shared Metabolic Pathway With Free Fucose

The comparison between the proteomes of two 2′FL concentrations of free fucose indicate close similarity between growth conditions (Figure 4B). Differential expression of FumD (Blon_RS11950) (log_2_ fold change 12.1, *p* = 0.004) and FumE (Blon_RS11965) (log_2_ fold change 14.0, *p* = 9.75E-6) are also observed during growth on 2% 2′FL as compared to lactose. Unlike the free fucose proteome, L-fucose permease (FucP) was not detected during growth on 2′FL. Thus, it is likely that there is a single metabolic pathway for fucose catabolism once fucose is translocated across the cell envelope by *B. infantis* ATCC 15697. This is based on expression of the same fucose-associated proteins regardless of conditions and variation in the specific transport proteins detected. During growth on 2% 2′FL, two orthologs of FumS (Blon_RS01790 and Blon_RS11370) show significantly increased expression relative to lactose (log_2_ fold change 12.6, *p* = 0.0004; 9.3, *p* = 0.005, respectively). FumS was not seen to be significantly differentially expressed during growth on fucose as compared to lactose. Interestingly, fucose may induce the cell to translocate 2′FL *via* dedicated ABC transporters, whereas 2′FL may not provide the converse signal to transport fucose through FucP.

### Varying 2′FL Concentration Does Not Significantly Alter the *B. infantis* Proteome

2′FL concentration impacts the *B. infantis* growth phenotype (Figure 3A) which is not fully reflected in normalized metabolite concentrations (Figures 3B,C). Using the proposed pathway in *B. kashowanohense* as a reference (11), two 2′FL concentrations yielded highly similar proteomes at 2% and 0.8% 2′FL as depicted in Figure 4B and Supplementary Figure 8, respectively. FumT2 provides a minor exception in that the 2′FL-associated transport permease was identified with high confidence in all 2% 2′FL replicates, and 2 of 3 at 0.8% 2′FL. It is likely that this is not biological variation, rather due to low abundance of the protein. Fucose metabolism-related proteins are not significantly differentially expressed during growth on 0.8% 2′FL as compared to growth on 2% 2′FL during mid-log phase. It cannot be excluded that varying 2′FL concentrations impact relative levels of expressed protein at later stages in growth, when the divergence of growth phenotypes is more pronounced.

### 2′FL Induces Fucose-Associated Gene Expression in the *B. infantis* Transcriptome

**Figure 5:**
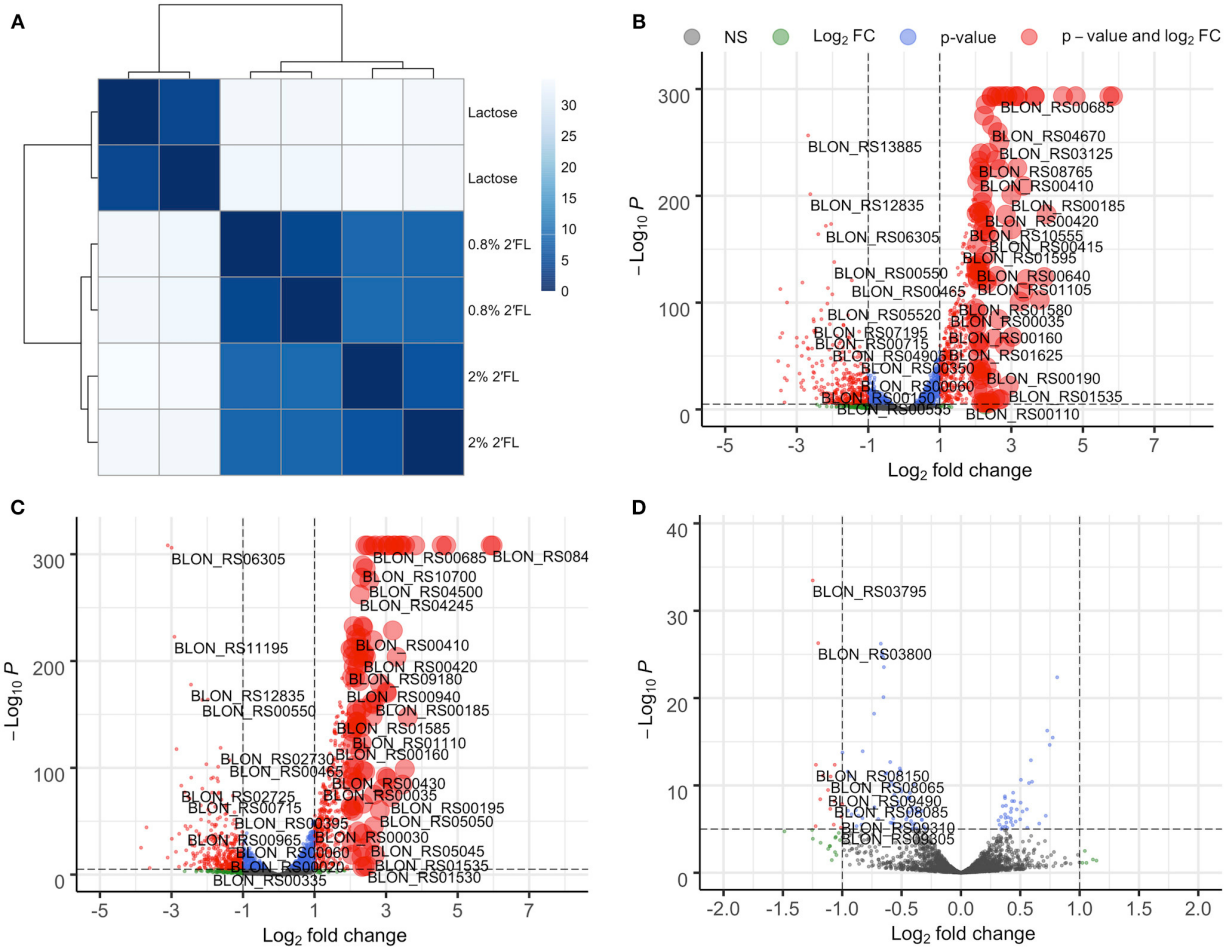
*B. infantis* gene expression is similar during growth on 0.8% and 2% 2′fucosyllactose as compared to during growth on lactose as seen from **(A)** sample-to-sample distance. While gene regulation is similar, there are several genes that show concentration-dependent regulation when volcano plots are used to compare **(B)** 0.8% 2′fucosyllactose and lactose, **(C)** 2% 2′fucosyllactose and lactose, and **(D)** 0.8% and 2% 2′fucosyllactose.

Regardless of 2′FL concentration (0.8 and 2%) the *B. infantis* ATCC 15697 transcriptomes are highly similar, as observed for their cognate proteomes. This is evidenced by low sample-to-sample distance between the two 2′FL concentrations and over two-fold higher sample-to-sample distance between 2′FL and lactose fermentations (Figure 5A) as well as clustering in the PCA plot (Supplementary Figure 9). Volcano plots confirm this observation as well (Figures 5B,C). There are relatively few genes differentially expressed between 2′FL concentrations (Figure 5D). Despite efforts to scale-up, the inefficient growth on free fucose did not generate sufficient quality and quantity of RNA for transcriptomics.

**Figure 6:**
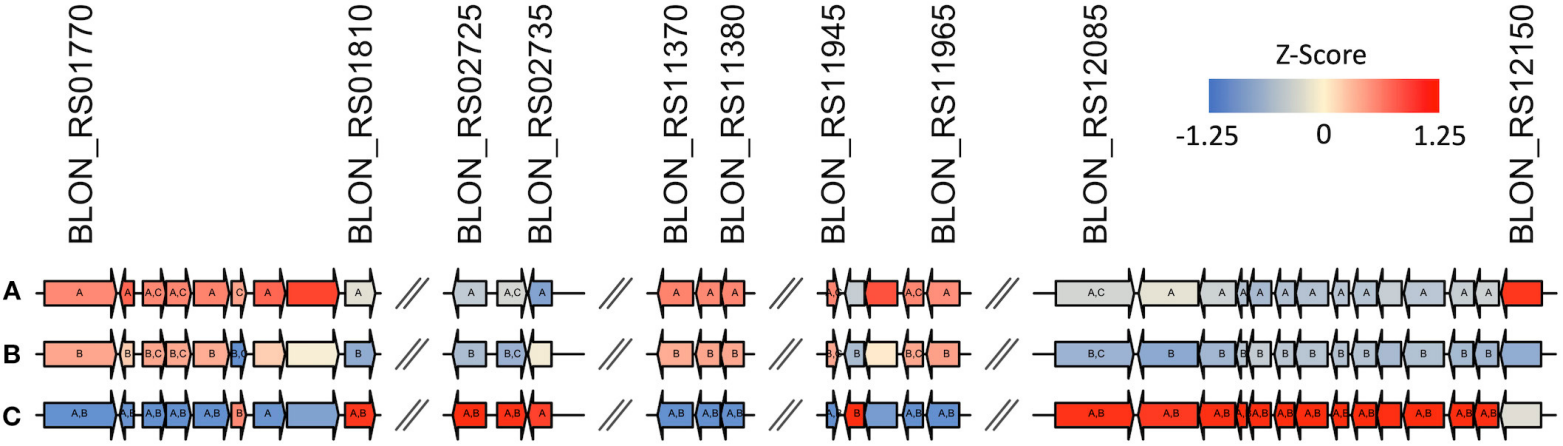
Gene maps show that there is differential regulation across the entire fucose metabolic pathway during growth on **(A)** 0.8% 2′fucosyllactose, **(B)** 2% 2′fucosyllactose, and **(C)** lactose as described by z-scores calculated from normalized count data. Gene annotations and predicted functions are provided in Supplementary Table 2.

Interestingly, 2′FL induces differential expression of the majority of proposed fucose metabolism genes relative to lactose (p ≤ 0.01; Figure 6, Supplementary Figures 10, 11). The exceptions are fuconate dehydratase (Blon_RS01795), fucose permease (Blon_RS11955), and solute-binding protein *fumS* (Blon_RS12150) which did not exhibit significant differences. These genes may not be expressed, or they may have similar constitutive expression levels between conditions. In addition, these three genes have multiple copies within the ATCC 15697 genome. Additional *fumE* paralogs are differentially expressed, with Blon_RS11965 highly upregulated (log_2_ fold change >4) whereas Blon_RS12115 is downregulated (log_2_ fold change < -1.5). Blon_RS01810 encodes another putative fucose permease which is downregulated (log_2_ fold change < -0.65, p ≤ 0.001). Blon_RS01790 (log_2_ fold change>3), Blon_RS11370 (log_2_ fold change>1.3), and Blon_RS12135 (log_2_ fold change < −1.4) encode *fumS*-like genes and are differentially regulated. There are several potential explanations for these observations, and they likely all contribute uncertainty. Foremost is that the putative *B. kashowanohense* pathway is incompletely characterized and validated including the constituent genes and their activities. Moreover, *B. infantis* fucose metabolism may diverge from *B. kashowanohense*, and paralogs may have multiple functions potentially unrelated to fucose metabolism.

Despite differential regulation between 2′FL relative to lactose, the transcriptomes remain relatively static regardless of 2′FL concentration (Figure 6). Two putative *fumS* genes (Blon_RS01790, *p* = 0.004 and Blon_RS12150, *p* = 0.002) and a *fumT1* gene (Blon_RS01785, *p* = 0.01) are down-regulated on 2% 2′FL relative to 0.8% 2′FL (Figure 6). This down-regulation may reflect the lower growth observed on higher concentrations of 2′FL. In addition, 2% 2′FL down-regulates a fucose dehydrogenase (Blon_RS11960; *p* < 0.01) and fuconate dehydratase (Blon_RS11965; *p* < 0.05). This down-regulation is consistent with lower growth on 2′FL. Similarly, a putative lactaldehyde reductase (Blon_RS02730; *p* < 0.0001) was downregulated during growth on 2% relative to 0.8% 2′FL. This enzyme could catalyze the conversion of lactaldehyde to 1,2-PD. Thus, differential regulation of fucose-related genes may reflect the non-linear relationship between 2′FL concentration and growth phenotype.

Differential regulation is not limited to the fucose-related genes previously identified. Given that the changes in metabolite production are not specific to fucose utilization (i.e., acetate, lactate, and formate) between 2′FL and lactose (Table 2), differential expression in the central pathway was examined. The majority of genes involved in the central pathway are up-regulated during growth on 2′FL as compared to lactose (Supplementary Figure 12). ROK family glucokinase Blon_RS02865 (*p* < 0.0001) and acetaldehyde-CoA/alcohol dehydrogenase Blon_RS11570 (*p* < 0.001) are both down-regulated during growth on 2′FL as compared to growth on lactose at both concentrations of 2′FL. Reduced glucokinase and acetaldehyde-CoA/alcohol dehydrogenase expression was also observed in *B. kashiwanohense* APCKJ1 during growth on 2′FL as compared to growth on both sorbitol and lactose (11). Genes implicated in sialic acid metabolism are similarly down-regulated in a consistent manner between both concentrations of 2′FL as compared to lactose (Supplementary Figure 13). This suggests that metabolism of the neutral fucosylated 2'FL regulates acidic HMO metabolism. It is unclear if this is a feature of all fucosylated HMOs or unique to the abundant 2'FL oligosaccharide species.

## Conclusions

*B. infantis* ATCC 15697 metabolizes both free and HMO-derived fucose through a common and likely phosphorylation-independent pathway to produce 1,2-PD. During fucose metabolism, carbon is shunted from lactate and toward formate and 1,2-PD production. This metabolic shift differs somewhat during free fucose metabolism relative to that of 2′FL. Although the inefficient metabolism of free fucose hinders direct comparisons in this respect. The shift to prioritize formate and 1,2-PD production may impact trophic relationships within the infant gut microbiome. Accordingly, it has been reported that the infant-associated *Eubacterium hallii* metabolizes 1,2-PD to produce short chain fatty acids butyrate, formate, and propionate. All of which potentially benefit the gut microbiome and overall infant development (52, 53). When presented with equal concentrations of fucose and lactose, *B. infantis* ATCC 15697 preferentially metabolizes the preferred lactose with minimal fucose utilization. Conversely, *B. infantis* ATCC 15697 exhibits significantly more growth on excess fucose with limiting concentrations of preferred carbohydrates, relative to individual carbohydrate components. It is clear that fucose catabolism is interconnected with the bifid shunt given the production of end-products typically secreted by bifidobacteria. The mechanistic relationship between the feeder fucose pathway and the bifid shunt is yet to be fully validated. Bifidobacterial metabolism of fucose may provide additional protection against enteric pathogens, which are known to utilize fucose cleaved from host mucin. Continued investigation of fucosylated HMOs and co-fermentation of free fucose in combination with other carbohydrate sources will enable greater clarity in this regard. Moreover, investigating additional *B. infantis* strains, as well as other species, will generalize observations to inform *in vitro* and *in vivo* host-microbial models in an effort to characterize metabolic networks between the infant and its microbiota. Ultimately, the goal is to understand this system with scientific rigor to potentially innovate precision nutrition interventions to support infant health and development.

## Data Availability Statement

Data are available *via* NCBI GEO under accession GSE159189 and *via* ProteomeXchange with identifier PXD021868.

## Author Contributions

LRD and DAS conceived, designed the study, wrote, revised, and approved the submitted manuscript. LRD, EÖ, and AR generated data. LRD, EÖ, AR, and DAS analyzed data. LRD drafted the initial manuscript. All authors contributed to the article and approved the submitted version.

## Conflict of Interest

DAS was on the scientific advisory board for Medolac Laboratories. The remaining authors declare that the research was conducted in the absence of any commercial or financial relationships that could be construed as a potential conflict of interest.
