# Supplementary material for: Bifidobacterium infantis Metabolizes 2′Fucosyllactose-Derived and Free Fucose Through a Common Catabolic Pathway Resulting in 1,2-Propanediol Secretion
Source: Front Nutr. 2020 Nov 24;7:583397. doi: 10.3389/fnut.2020.583397 (PMC7732495; doi:10.3389/fnut.2020.583397)
Supplement: Supplementary file 1 [file Data_Sheet_1.PDF]

Supplementary Material

***Bifidobacterium infantis* metabolizes 2´fucosyllactose-derived and free fucose through a common catabolic pathway resulting in 1,2-propanediol secretion**

Liv R. Dedon[1], Ezgi Özcan[1], Asha Rani[1], & David A. Sela[1,2,3*]

[1]Department of Food Science, University of Massachusetts Amherst, Amherst, MA United States
[2]Department of Microbiology, University of Massachusetts Amherst, Amherst, MA, United States
[3]Department of Microbiology and Physiological Systems and Center for Microbiome Research, University of Massachusetts Medical School, Worcester, MA, United States

[*]**Correspondence:**
David A. Sela
davidsela@umass.edu

Keywords: bifidobacteria, human milk oligosaccharides, fucose, 2´fucosyllactose, microbiota, microbiome

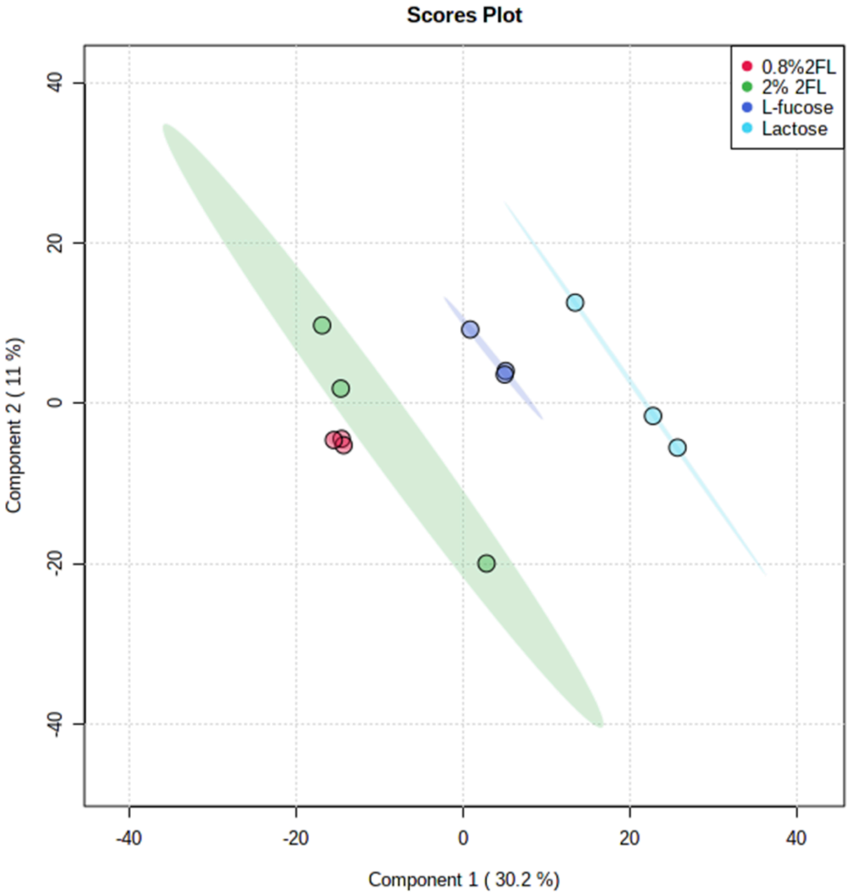

Figure S1: PLS-DA score plot for the different carbohydrate sources. Ellipses showing 95% confidence limits of a normal distribution for each group of the samples (three replicates were used for each group). Color legends are shown to the right for each group.

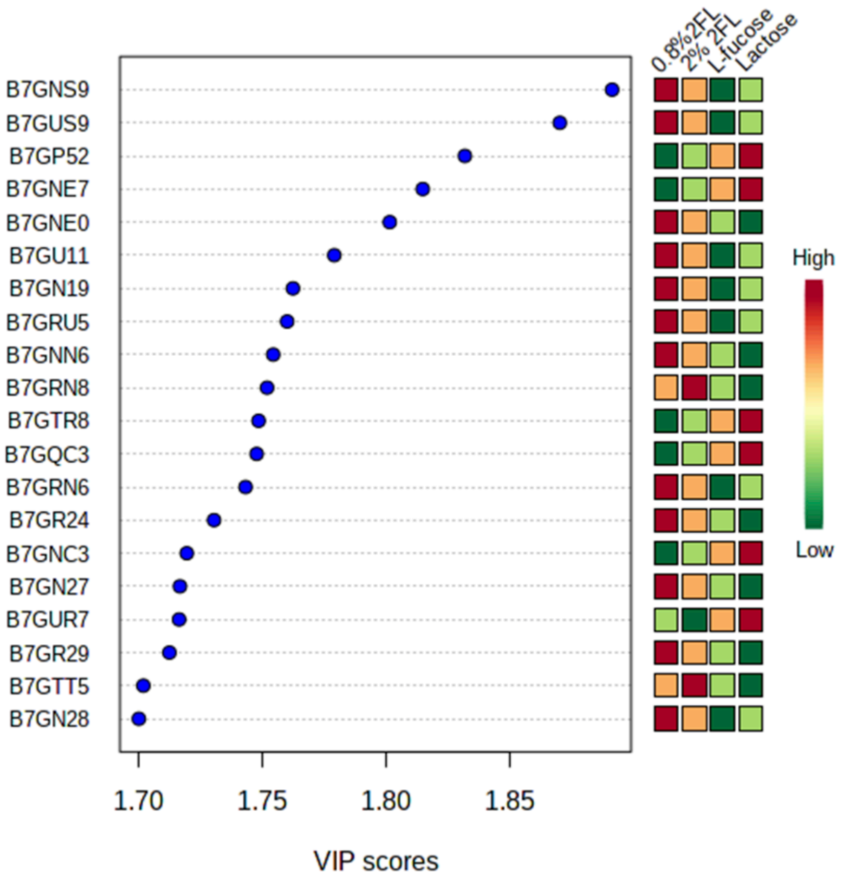

Figure S2: VIP (Variable Importance in Projection) scores obtained after pair-wise PLS-DA analysis for (A) 0.8% 2′FL, (B) 2% 2′FL, (C) fucose, and (D) lactose. A VIP score of ≥1.0 is considered significant. The colored boxes on the right indicate the relative abundance of the corresponding proteins in each group.

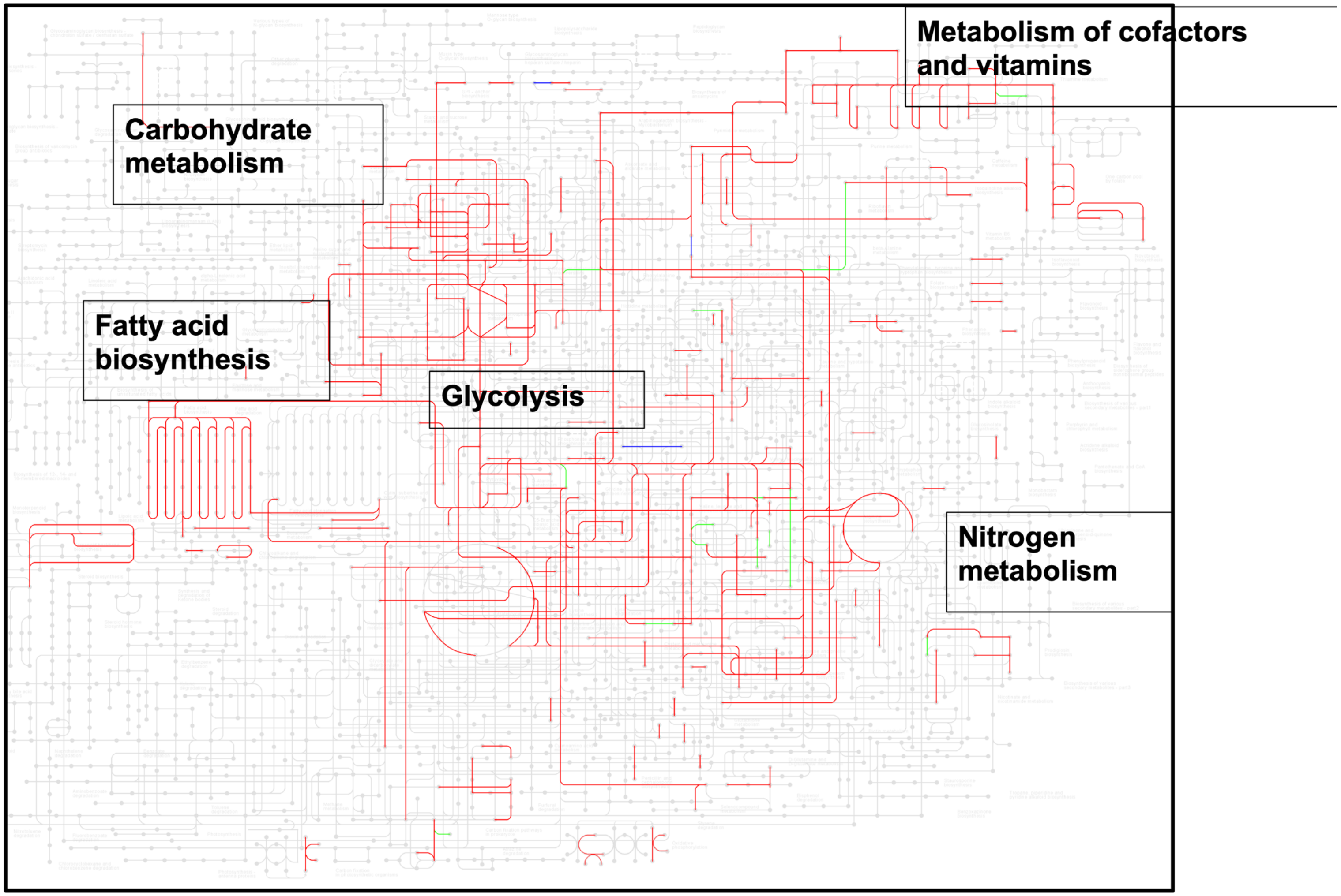

Figure S3: KEGG metabolic pathway map of the *B. infantis* proteome during growth on 0.8% and 2% 2′FL as the sole carbohydrate source. Proteins which are identified in both datasets (0.8% and 2% 2′FL) are marked in red, only in 0.8% 2′FL in blue, and only in 2% 2′FL are marked in green in the metabolic pathways.

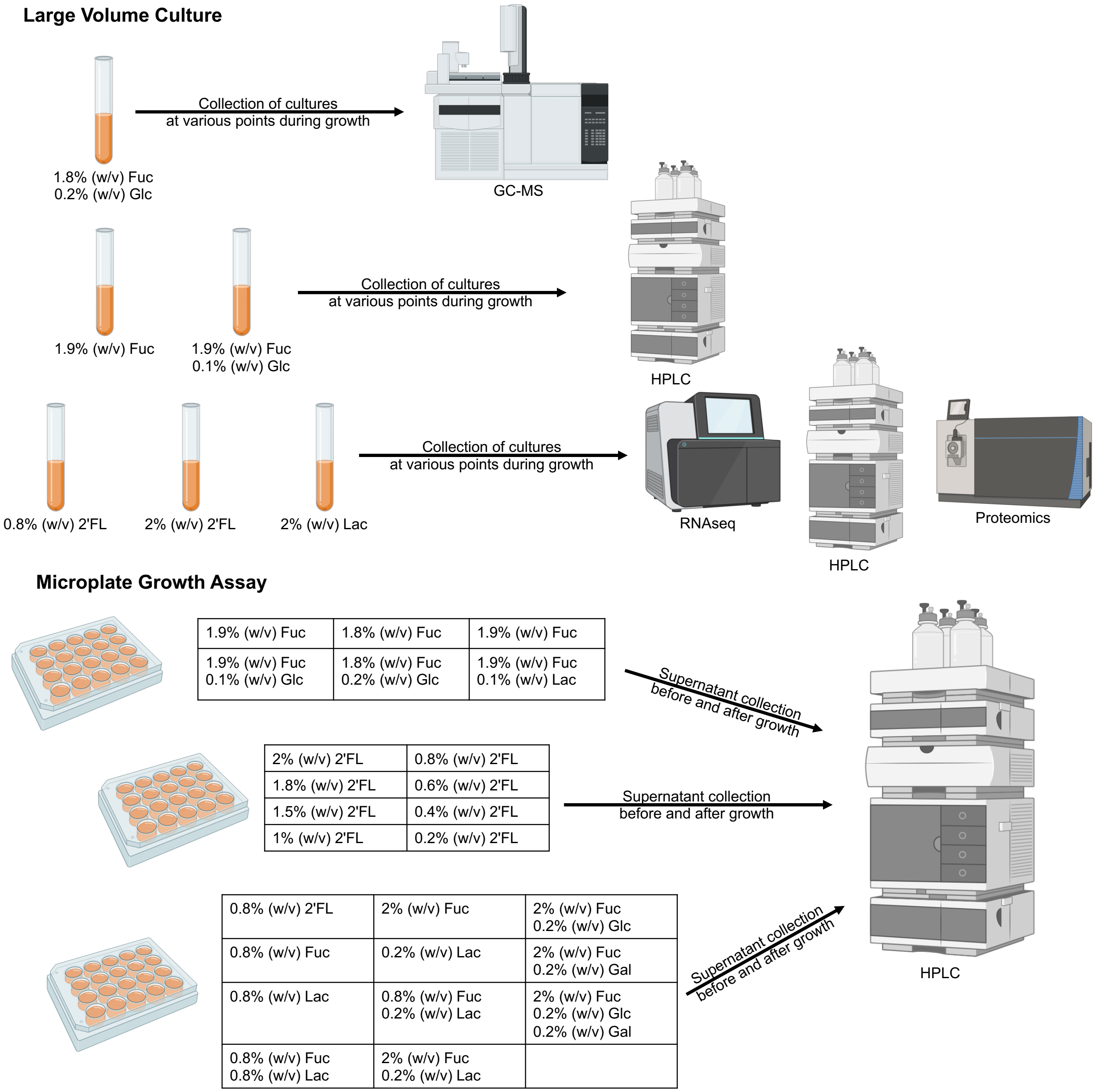

Figure S4: Schematic of culture experiments performed in this study.

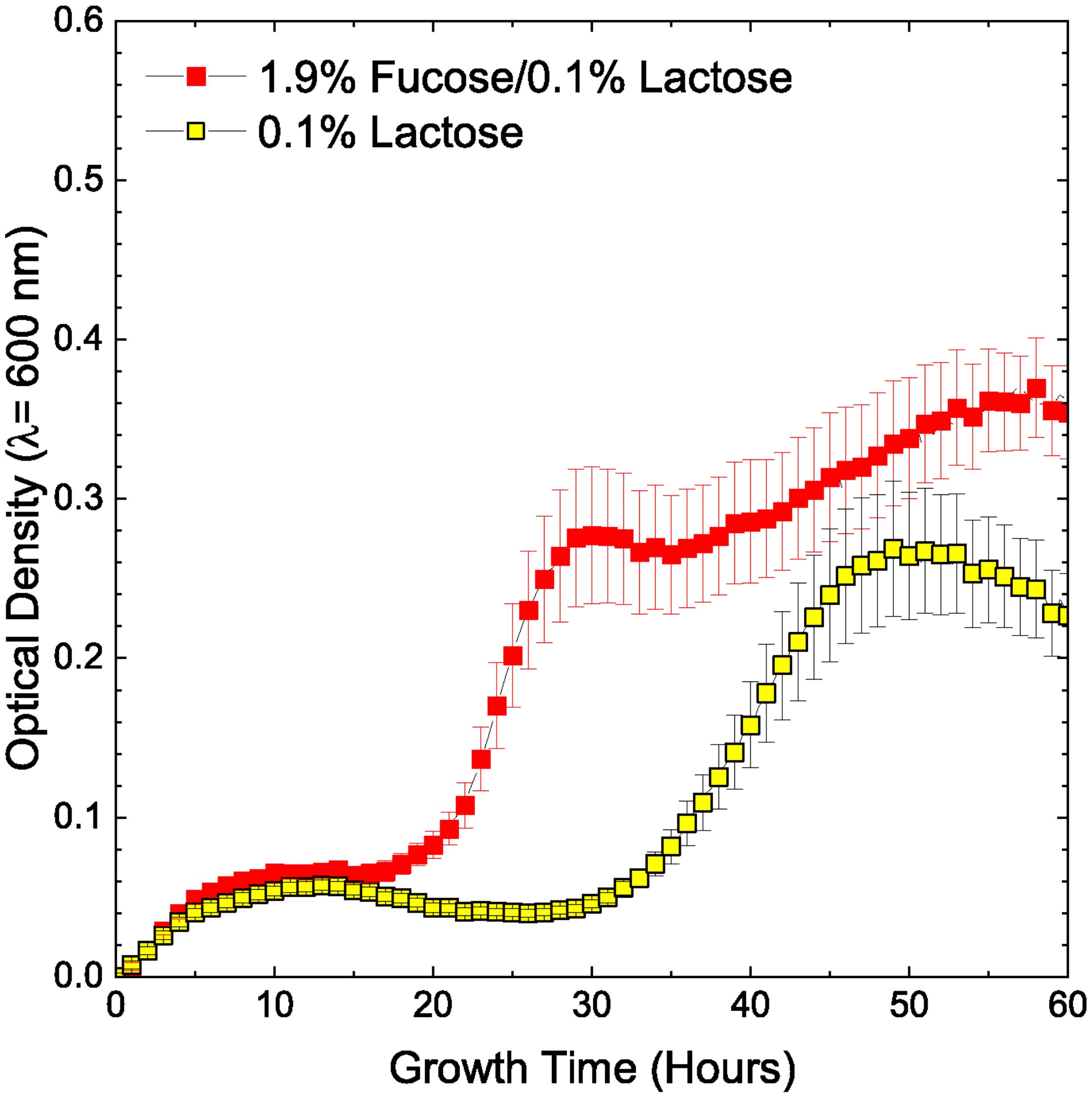

Figure S5: *B. infantis* growth behavior during growth in co-fermentation of fucose and lactose is not significantly different than on the corresponding concentration of lactose as a sole carbohydrate source.

Tabls S1: Metabolite production during *B. infantis* growth in co-fermentation of fucose and lactose suggests that fucose metabolism is limited.

	Lactose (mM)	Fucose (mM)	Lactate (mM)	Formate (mM)	Acetate (mM)	1,2PD (mM)	Ace:Lac	Form:Ace
1.9% Fucose/ 0.1% Lactose	-6.0±0.5	-6±5	+4.8±0.4	+8±1	+17±1	+8.3±0.5	2.2	0.60
1.9% Fucose	ND	-23±4	+4.2±0.3	+16.4±0.8	+23±5	+13.1±0.3	2.84	0.82
0.1% Lactose	-5.2±0.8	ND	+3.2±0.3	+4±1	+13.7±0.5	ND	2.7	0.33

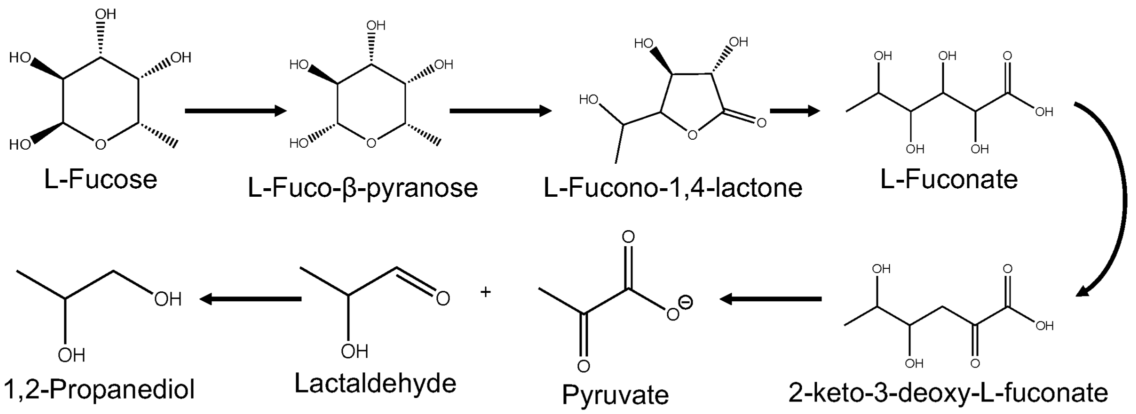

Figure S6: The proposed bifidobacterial fucose metabolic pathway

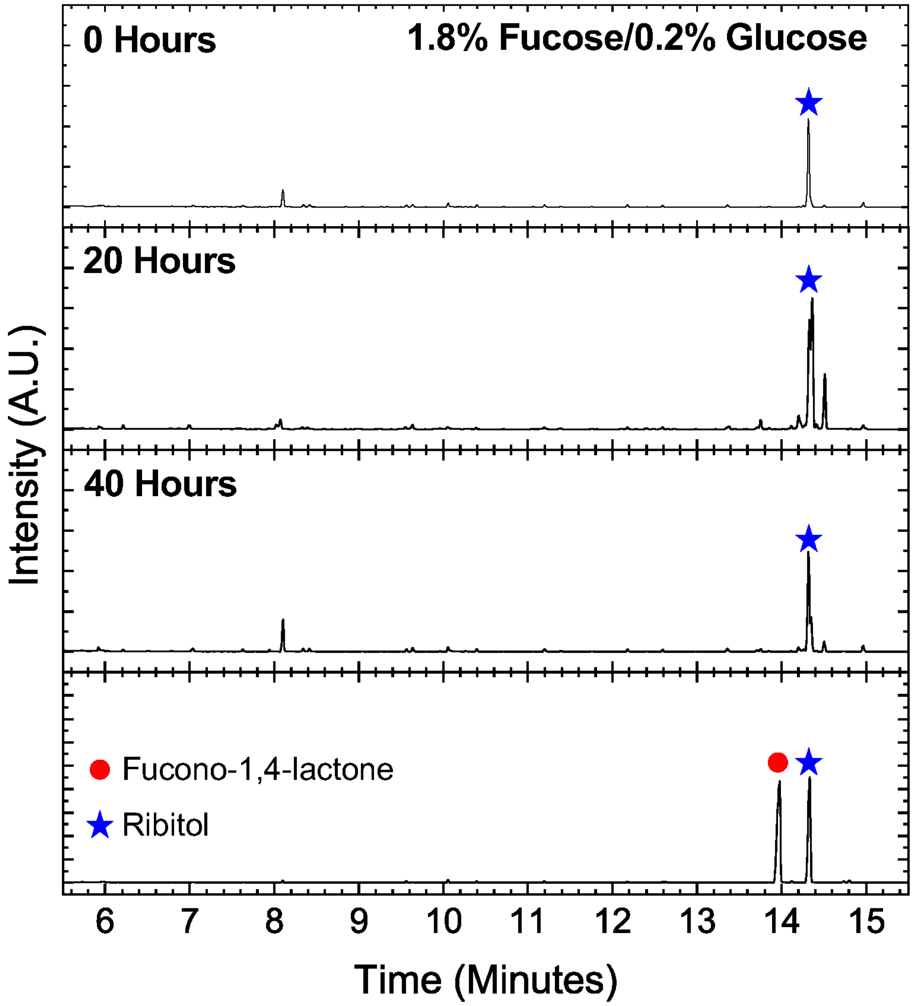

Figure S7: Fucose-specific intracellular metabolite fucono-1,4-lactone is not observed in gas chromatograms at any point during growth in co-fermentation of 1.8% fucose and 0.2% glucose.

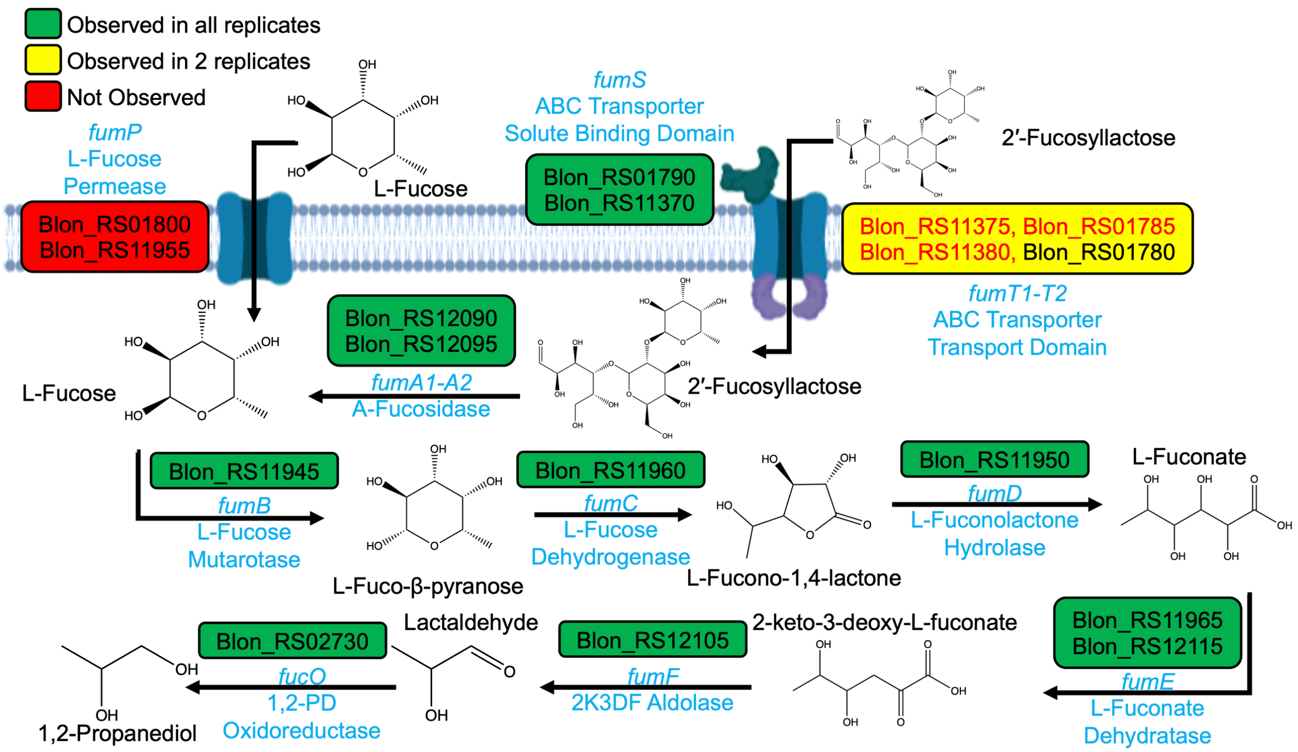

Figure S8: Proposed fucose pathways based on proteome during growth on 0.8% 2′fucosyllactose as sole carbohydrate source. Gene symbols are given in light blue above the reaction arrows and corresponding protein names below.

Table S2: Gene annotations, predicted functions, and protein KEGG orthology for the predicted *B. infantis* fucose metabolic pathway.

Gene ID	Gene Name	Protein KO	Function
BLON_RS01770			ATP-dependent DNA helicase
BLON_RS01775	*fumR*	K03655	LacI transcriptional regulator
BLON_RS01780	*fumT2*	K02025	ABC transporter permease
BLON_RS01785	*fumT1*	K02026	Binding-protein-dependent transport systems inner membrane component
BLON_RS01790	*fumS*	K02027	Extracellular solute-binding protein, family 1
BLON_RS01795	*fumE*	K18334	L-fuconate dehydratase
BLON_RS01800			Major facilitator, superfamily MFS_1
BLON_RS01805			Hypothetical protein
BLON_RS01810	*fucP*	K02429	L-fucose permease
BLON_RS02725		K00574	Cyclopropane-fatty-acyl-phospholipid synthase
BLON_RS02730	*fucO*	K00048	L-1,2-propanediol oxidoreductase
BLON_RS02735			Glycosyl transferase, family 2
BLON_RS11370	*fumS*	K02027	Extracellular solute-binding protein, family 1
BLON_RS11375	*fumT1*	K02026	Carbohydrate ABC transporter permease
BLON_RS11380	*fumT2*	K02025	ABC transporter permease
BLON_RS11945	*fumB*	K02431	L-fucose mutarotase
BLON_RS11950	*fumD*		L-fuconolactone hydrolase
BLON_RS11955	*fucP*	K02429	L-fucose permease
BLON_RS11960	*fumC*	K18333	L-2- keto-3-deoxy-fuconate-4-dehydrogenase/L-fucose dehydrogenase
BLON_RS11965	*fumE*	K18334	L-fuconate dehydratase
BLON_RS12085		K01190	Glycoside hydrolase, family 2, TIM barrel
BLON_RS12090	*fumA1*	K15923	GH95 alpha-1 3/4-fucosidase
BLON_RS12095	*fumA2*	K01206	GH29 alpha-1 3/4 fucosidase
BLON_RS12100	*fumB*	K02431	L-fucose mutarotase
BLON_RS12105	*fucA*	K22397	L-2-keto-3-deoxy-fuconate aldolase
BLON_RS12110	*fumC*	K18333	L-fucose dehydrogenase
BLON_RS12115	*fumE*	K18334	L-fuconate dehydratase
BLON_RS12120			Hypothetical protein
BLON_RS12125	*fumT1*	K02026	ABC transporter permease
BLON_RS12130		K17319	ABC transporter permease
BLON_RS12135	*fumS*	K02027	Extracellular solute-binding protein, family 1
BLON_RS12140	*fumT1*	K02026	ABC transporter permease
BLON_RS12145		K17319	ABC transporter permease
BLON_RS12150	*fumS*	K02027	Extracellular solute-binding protein, family 1

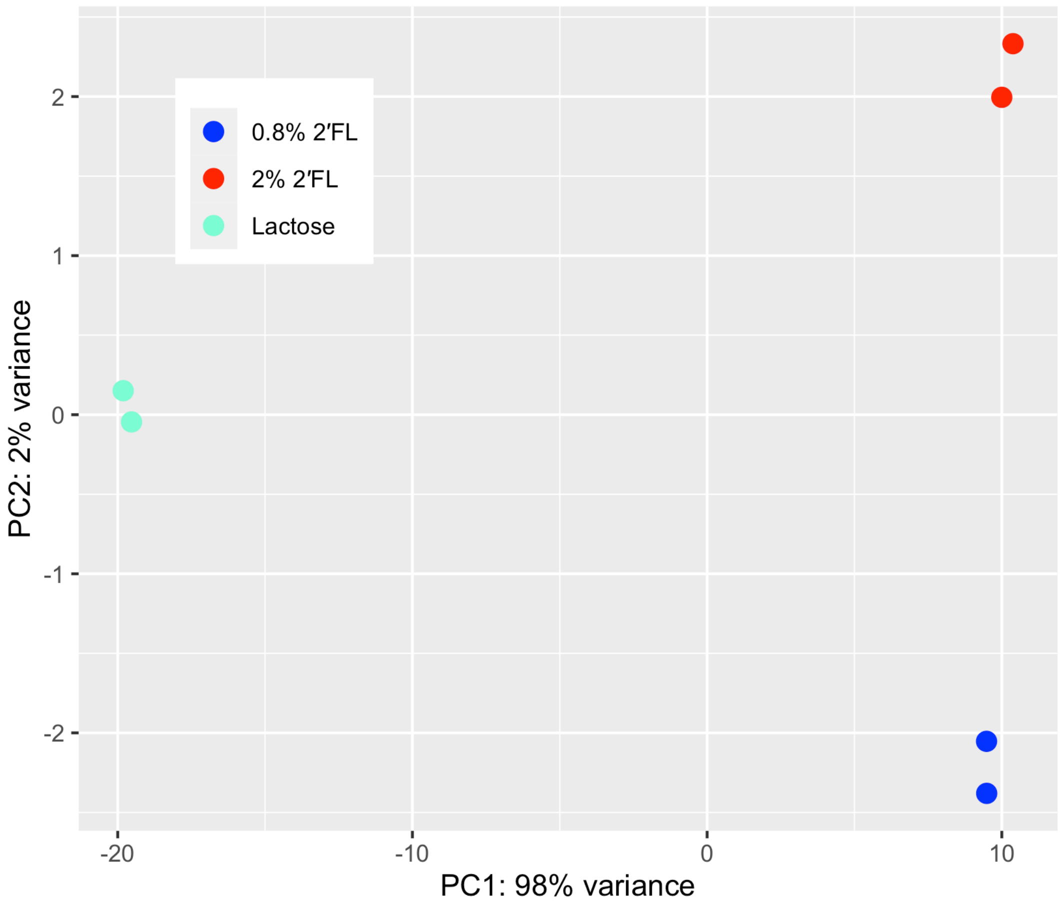

Figure S9: Principal component analysis of the *B. infantis* transcriptome exhibits low variance during growth on 2% and 0.8% 2′fucosyllactose as sole carbohydrates but suggests that transcription during growth on lactose as a sole carbohydrate varies.

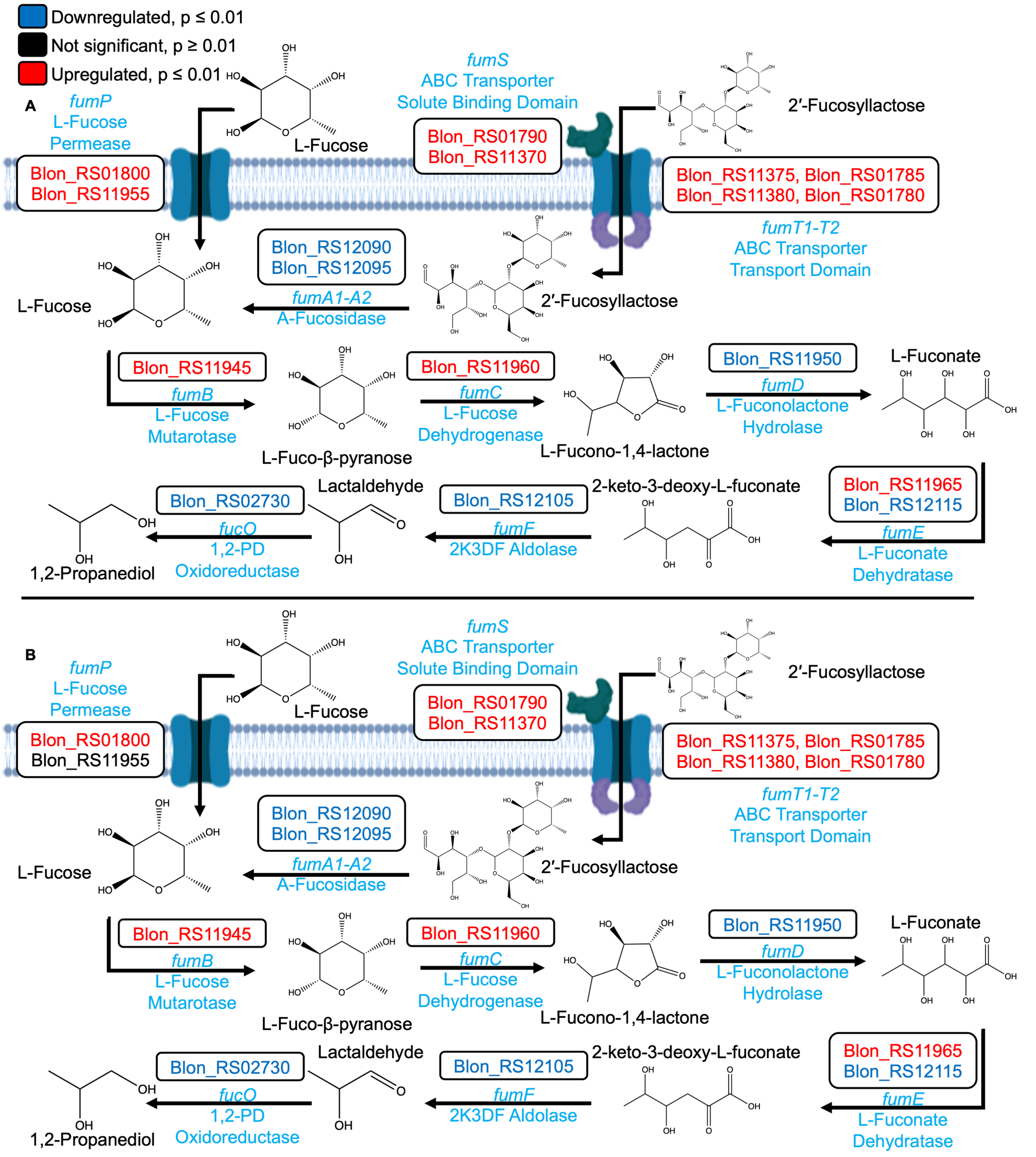

Figure S10: Proposed fucose pathways based on the transcriptome during growth on (A) 0.8% and (B) 2% 2′fucosyllactose as sole carbohydrate sources. Gene symbols are provided in light blue above the reaction arrows and corresponding protein names below. Upregulation is defined as log2foldchange > 0, and downregulated as log2foldchange < 0.

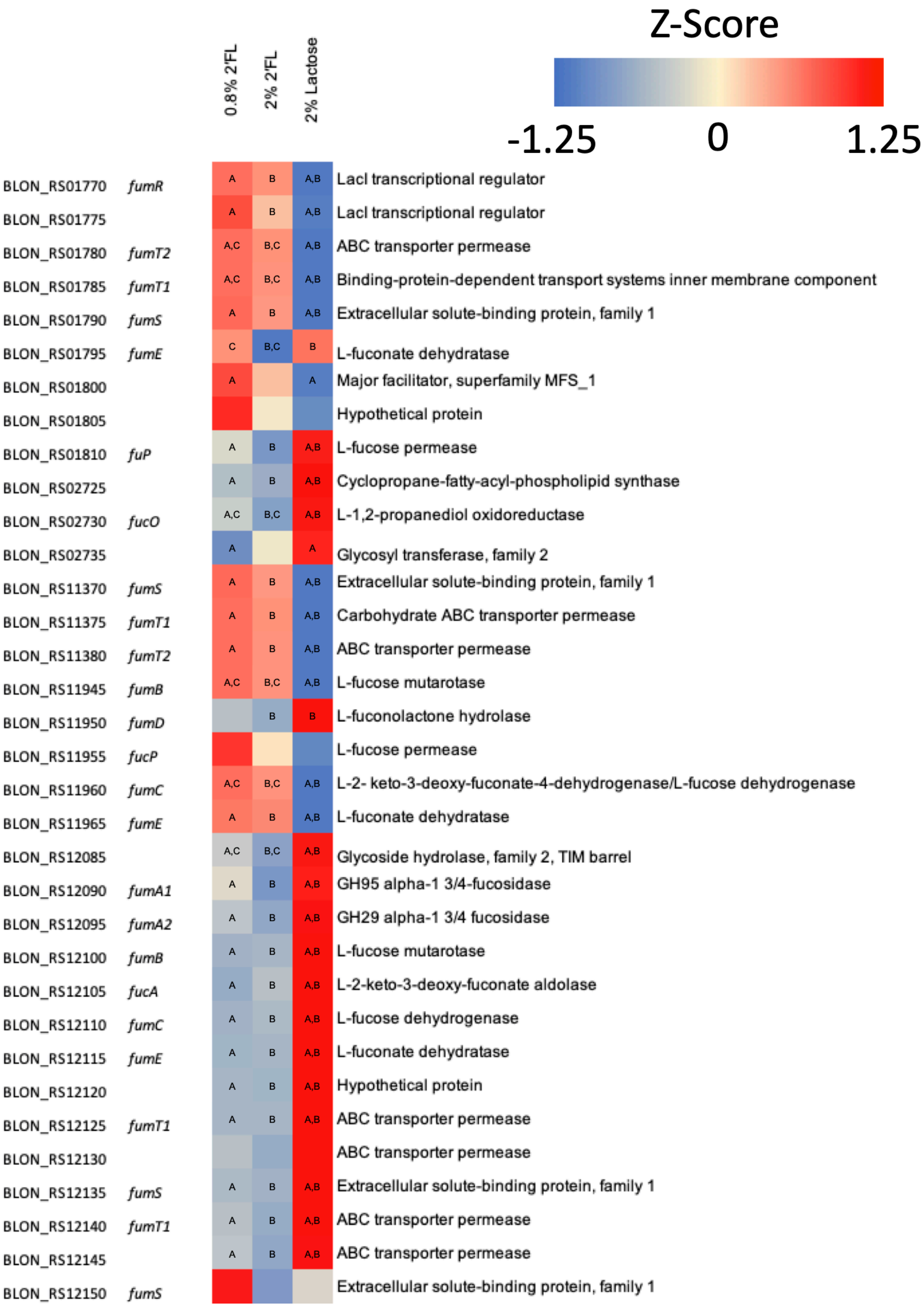

Figure S11: *B. infantis* exhibits differential expression of genes involved in the fucose metabolic pathway as described by z-score calculated from normalized count data. Significance of $p \leq 0.5$ is denoted as A, between 0.8% 2′FL and lactose; B, between 2% 2′FL and lactose; and C, between 0.8% and 2% 2′FL.

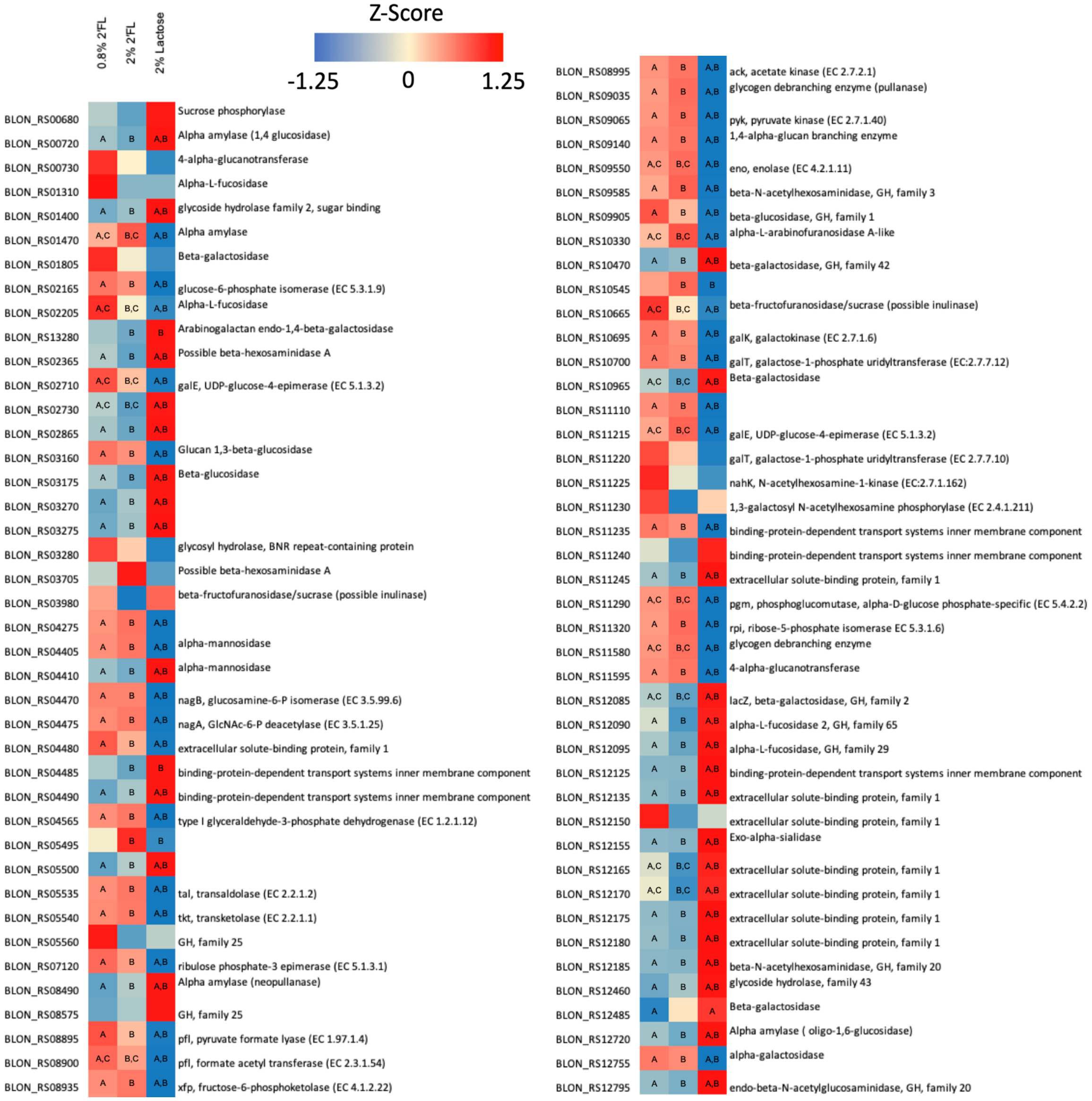

Figure S12: *B. infantis* exhibits differential expression of genes involved in central metabolic processes of the bifid shunt as described by z-score calculated from normalized count data. Significance of $p \leq 0.5$ is denoted as A, between 0.8% 2′FL and lactose; B, between 2% 2′FL and lactose; and C, between 0.8% and 2% 2′FL.

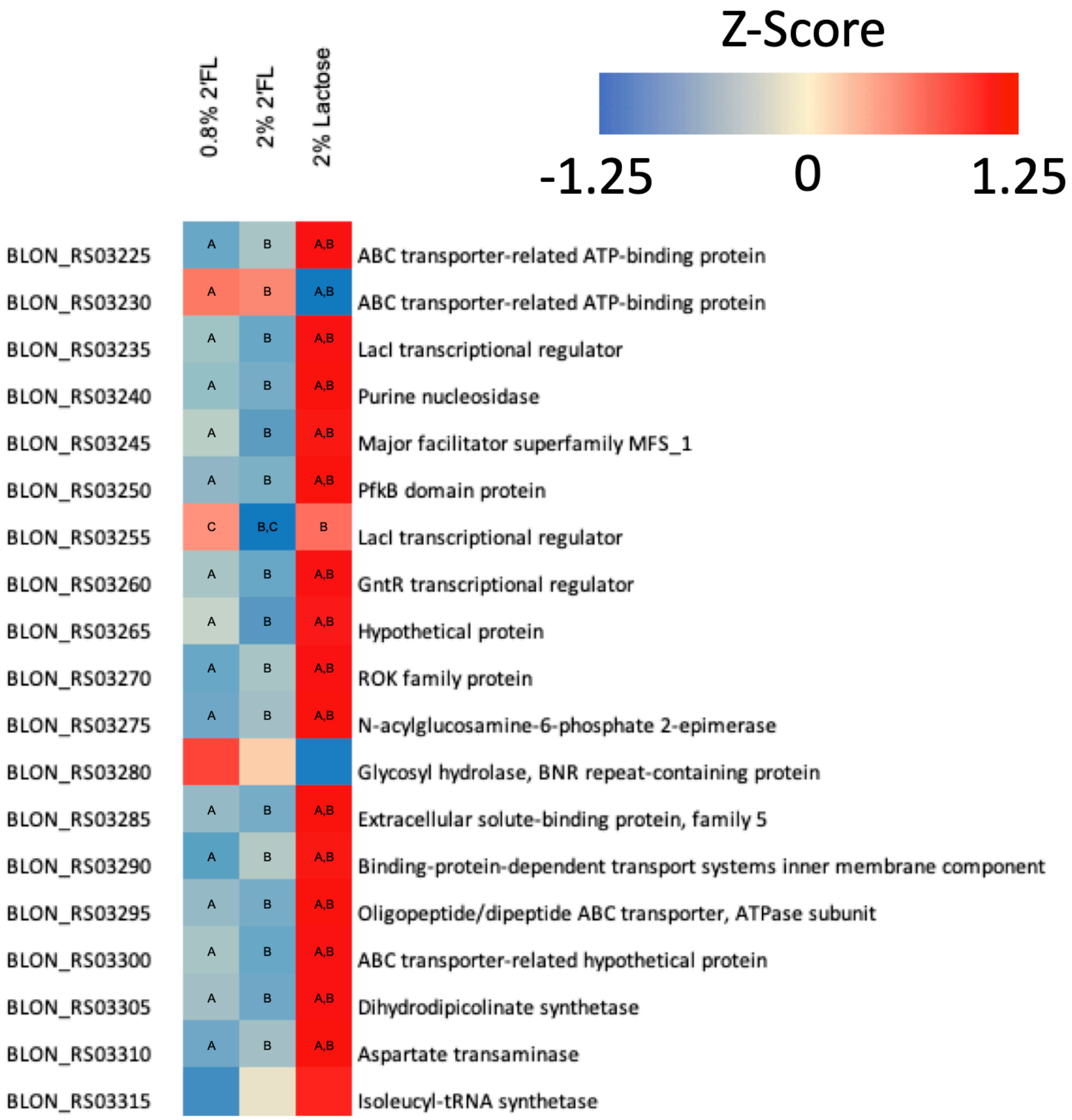

Figure S13: *B. infantis* exhibits differential expression of genes involved in sialic acid metabolism as described by z-score calculated from normalized count data. Significance of $p \leq 0.5$ is denoted as A, between 0.8% 2′FL and lactose; B, between 2% 2′FL and lactose; and C, between 0.8% and 2% 2′FL.
